# Hierarchical porous ECM scaffolds incorporating GDF-5 fabricated by cryogenic 3D printing to promote articular cartilage regeneration

**DOI:** 10.1186/s40824-023-00349-y

**Published:** 2023-02-05

**Authors:** Jiang Wu, Liwei Fu, Zineng Yan, Yu Yang, Han Yin, Pinxue Li, Xun Yuan, Zhengang Ding, Teng Kang, Zhuang Tian, Zhiyao Liao, Guangzhao Tian, Chao Ning, Yuguo Li, Xiang Sui, Mingxue Chen, Shuyun Liu, Quanyi Guo

**Affiliations:** 1 Guizhou Medical University, Guiyang, 550004 Guizhou Province People’s Republic of China; 2 Beijing Key Laboratory of Regenerative Medicine in Orthopedics; Key Laboratory of Musculoskeletal Trauma & War Injuries PLA, Institute of Orthopedics, Chinese PLA General Hospital, No. 28 Fuxing Road, Haidian District, Beijing, 100853 People’s Republic of China; 3 School of Medicine, Nankai University, Tianjin, 300071 People’s Republic of China; 4 Department of Orthopedics, The Second People’s Hospital of Guiyang, 547 Jinyang South Road, Guiyang, 550023 Guizhou China; 5 Department of Orthopaedic Surgery, Peking University Fourth School of Clinical Medicine, Beijing Jishuitan Hospital, No. 31 Xinjiekou East Street, Xicheng District, Beijing, 100035 People’s Republic of China

**Keywords:** Decellularized cartilage extracellular matrix, Low-temperature deposition manufacturing, Tissue engineering, Articular cartilage regeneration

## Abstract

**Background:**

In recent years, there has been significant research progress on in situ articular cartilage (AC) tissue engineering with endogenous stem cells, which uses biological materials or bioactive factors to improve the regeneration microenvironment and recruit more endogenous stem cells from the joint cavity to the defect area to promote cartilage regeneration.

**Method:**

In this study, we used ECM alone as a bioink in low-temperature deposition manufacturing (LDM) 3D printing and then successfully fabricated a hierarchical porous ECM scaffold incorporating GDF-5.

**Results:**

Comparative in vitro experiments showed that the 7% ECM scaffolds had the best biocompatibility. After the addition of GDF-5 protein, the ECM scaffolds significantly improved bone marrow mesenchymal stem cell (BMSC) migration and chondrogenic differentiation. Most importantly, the in vivo results showed that the ECM/GDF-5 scaffold significantly enhanced in situ cartilage repair.

**Conclusion:**

In conclusion, this study reports the construction of a new scaffold based on the concept of in situ regeneration, and we believe that our findings will provide a new treatment strategy for AC defect repair.

**Supplementary Information:**

The online version contains supplementary material available at 10.1186/s40824-023-00349-y.

## Introduction

Articular cartilage (AC) plays a crucial role in maintaining joint motion. However, due to its limited regeneration capacity, AC damage caused by sports injury, trauma and ageing may eventually lead to osteoarthritis (OA) [1, 2]. Currently, the long-term prognostic effects of common therapeutic strategies, including arthroscopic debridement, microfracture (MF), or autologous chondrocyte implantation (ACI), are still unsatisfactory [3]. In recent years, research on in situ AC tissue engineering techniques with endogenous stem cells has made good progress [4]. The main factors affecting AC in situ repair include the destruction of the regenerative microenvironment in the defect area and an insufficient number of stem cells [5, 6]. In situ tissue engineering uses biological materials or bioactive factors to improve the regeneration microenvironment and recruit more endogenous stem cells from the joint cavity to the defect area to promote cartilage regeneration [7].

Among many biomaterials, decellularized cartilage extracellular matrix (ECM) has been widely used because of its outstanding biocompatibility and biochemical cue retention [8, 9]. Previous studies by our group have also demonstrated the positive effect of ECM on cartilage regeneration [10, 11]. However, traditional ECM scaffolds are usually lyophilized. In addition to their poor mechanical properties, their uneven pore size affects further ECM application. As a representative technology for precise scaffold fabrication, 3D printing is widely used in the construction of cartilage tissue engineering scaffolds [12, 13]. However, traditional high-temperature-melt fused deposition modelling (FDM) printing technology usually destroys the chemical structure of the material, leading to a capacity reduction [14]. Low-temperature deposition manufacturing (LDM) provides a possibility for scaffold fabrication while retaining the biological activity of the materials [15]. Unlike in FDM, bioinks are ejected from the printing nozzle and immediately solidify on a cryogenic platform [16]. After lyophilization, the bioinks form a hierarchical porous structure [17]. The interconnected porous structure and high porosity of scaffolds are not only essential for cell adhesion and growth but are also beneficial for oxygen and nutrient diffusion [15, 17]. In particular, recent studies have shown that scaffolds supported with macropores and micro/nanostructures perform better than only macroporous scaffolds in vivo [17, 18]. This hierarchical structure provides more adsorption sites for bioactive molecules and improves nutrient and metabolic waste transport. Chen et al. mixed ECM and waterborne polyurethane (WPU) to make bioinks for cryogenic 3D printing, constructed a hybrid scaffold and showed excellent repair effects in vivo [14]. However, the addition of synthetic materials still destroys the biochemical cues of the ECM itself, and making pure ECM bioink and printing it into scaffolds using LDM seems to better preserve its active properties. In addition, to show the advantage of the new scaffold (LDM) over the traditional scaffold (FDM), we added the FDM structure group to the experimental groups compared in the Supplementary File.

An ideal cartilage tissue-engineered scaffold must be biocompatible and porous while also having sufficient biological stimulation [19, 20]. Therefore, to improve overall scaffold performance, we need to supplement the corresponding bioactive factors. GDF-5, a member of the TGF-β/BMP superfamily, is essential for prechondrogenic mesenchymal stem cell (MSC) cohesion and directly affects cartilage and bone development [21, 22]. Recently, some studies have shown that GDF-5 protein significantly promotes bone marrow mesenchymal stem cell (BMSC) migration and chondrogenic differentiation [23, 24]. Therefore, GDF-5 was introduced to improve the scaffold recruitment of MSCs and chondrogenic differentiation.

**Fig. 1:**
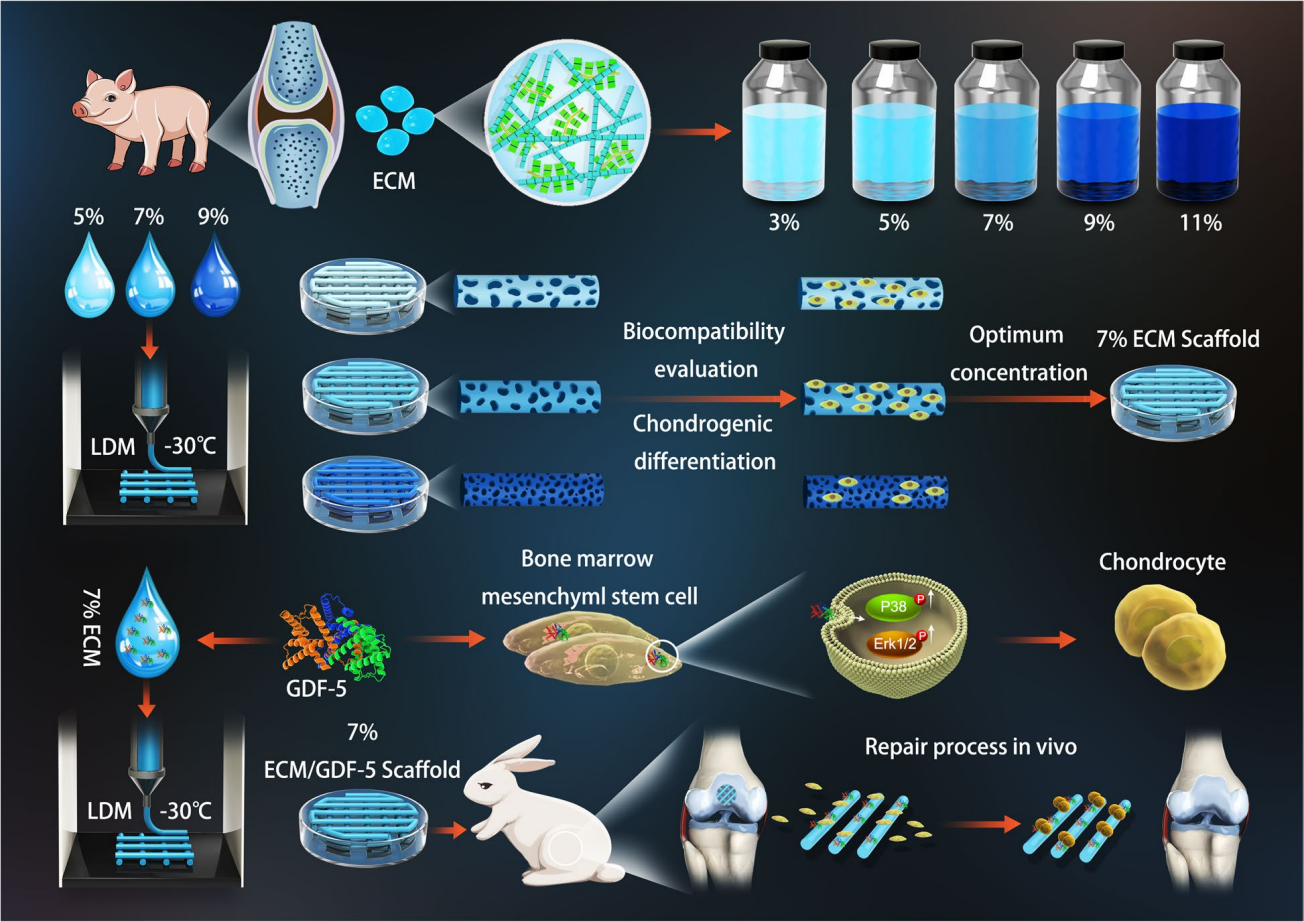
Schematic illustration of the study design

As shown in Fig. 1, we first prepared a pure ECM bioink with an optimal concentration, which was then mixed with GDF-5 and coprinted by LDM to fabricate a hierarchical porous scaffold. The functional scaffold not only provided the necessary microenvironment for articular cartilage regeneration but also promoted more MSC migration to the defect area and further enhanced chondrogenic differentiation through sustained release of GDF-5. Furthermore, we also evaluated the regenerative potential of the hierarchical porous ECM/GDF-5 scaffold in vivo and discussed its prospects for future application. To the best of our knowledge, this is the first report on the construction of cartilage tissue engineering scaffolds by LDM using pure ECM as the main bioink component, and we believe that our findings will provide a new treatment strategy for AC defect repair.

## Materials and methods

### Preparation and characterization of ECM bioinks

#### Preparation of ECM bioinks

Decellularized cartilage ECM was prepared by a combination of chemical and physical methods based on previous studies with slight modifications [10, 11, 25]. The specific experimental procedure is detailed in Supplementary File section 1.1. The ECM homogenate was lyophilized and then broken and ground into dry powder, and then 3, 5, 7, 9, and 11% (mass/volume) ECM was selected for preliminary bioink exploration. Specifically, 3.0 g, 5.0 g, 7.0 g, 9.0 g, and 11.0 g of dried cartilage ECM was weighed and added to beakers containing 100 ml of 5% acetic acid solution. Next, ECM bioinks at different concentrations were obtained by crushing the material in an ice bath at 75% amplitude for a total of 2 min using a Q125 ultrasonograph (Qsonica, USA).

#### Gelation status and particle size of ECM bioinks

The gelation status of ECM bioinks at different concentrations was assessed by a dispensing needle to examine the printability.

To further evaluate the printability of ECM bioinks, we examined their particle size using dynamic light scattering (Delsa™ Nano C Particle Analyser).

#### Fourier transform infrared (FTIR) spectroscopy

The functional groups of the lyophilized ECM bioinks at different concentrations were identified using a Bruker Tensor 27 FTIR spectrometer (Nicolet IS5, Germany). The samples were tested in reflection mode. All spectra were recorded between 4000 and 500 cm ^− 1^ with a resolution of 1 cm ^− 1^.

#### Rheological characterization of ECM bioinks

As a crucial indicator for assessing the printability of bioinks, the rheological properties of ECM bioinks were tested with a Paar MCR 301 advanced rotational rheometer (Austria) with a diameter of 20 mm. The storage and loss moduli of bioinks were tested at 25 °C at 101-103 Hz. The shear viscosity of the bioinks was measured at different concentrations at an angular velocity of 101-102.

### Preparation and characterization of pure ECM scaffolds

#### Preparation of pure ECM scaffolds

We prepared pure ECM scaffolds with different ECM concentrations by an LDM 3D printing system. The abovementioned ECM bioinks at concentrations of 5, 7, and 9%, which were suitable for printing, were loaded into a 3D printer fitted with a plastic syringe with ink and equipped with a low-temperature receiver plate. Briefly, the printer followed a predesigned printing route for the scaffold model, and the bioink was layered through the printhead and deposited on a cryogenic receiver plate at − 30 °C. Then, the extruded fibre diameter was quickly cured during the printing process. All printed scaffolds were transferred to − 80 °C and lyophilized in a freeze dryer for 48 hours to remove residual solvents. Finally, all scaffolds were sterilized by cobalt 60 and used for subsequent biological experiments.

#### Scanning Electron Microscopy (SEM) and particle size distribution

To evaluate the microstructures of the ECM scaffolds with different ECM concentrations, we observed them by scanning electron microscopy (SEM, S-4800, Hitachi, Japan). In brief, the freeze-dried ECM scaffolds were fixed and sprayed with gold. The microstructures were observed and photographed under an electron microscope, and the micropore size distributions of the scaffolds were further analysed by ImageJ software.

#### Porosity measurement and biomechanical assay

To measure the porosity of the scaffolds, the classical ethanol replacement method was used in this study. Detailed experimental procedures are available in Supplementary File section 1.2.

To evaluate the biomechanical properties of the ECM scaffolds with different ECM concentrations, 5*5*1.2 mm^3^ scaffolds were prepared, with 3 scaffolds per concentration. The mechanical strength of the scaffold was tested using a BOSE biomechanical testing machine (BOSE 5100, USA). We assessed the compressive strength of the scaffolds by plotting a strain–stress curve, and the compression modulus was defined according to the linear slope of the strain–stress curve.

### In vitro cytocompatibility studies of pure ECM scaffolds

#### Isolation, culture and identification of BMSCs

This study was approved by the Animal Ethics Committee of the General Hospital of the Chinese People’s Liberation Army. BMSC isolation and culture were performed according to a previously described method [26, 27], and the specific steps are described in Supplementary File section 1.3.

Then, we used flow cytometry (Beckman Coulter, CytoFLEX) to identify BMSCs. Antibodies for positive surface markers included CD 105-FITC (NB500-452PECY7) and CD 90-PE (BD Biosciences, 561,409), while negative markers included CD 34-PE (Novus, NB2-54355F) and CD 45-PE (BD Biosciences, 561,624).

#### Cell viability and proliferation assay

BMSCs were seeded on the ECM scaffolds to assay their viability on scaffolds with different ECM concentrations. After seeding, DMEM was added, and the cells were cultured for 7 days. The medium was changed every 2 days, and then the cells on the scaffolds were stained using a live/dead assay kit. Specifically, the scaffolds were washed with PBS and stained using 100 μl of a double staining solution containing 2 μmol of calcein-am and 4 μmol of propidium iodide. Subsequently, the numbers of live and dead cells on the ECM scaffolds were observed using a laser confocal microscope (Nikon, Tokyo, Japan).

The CCK-8 assay was performed to detect the effects of the ECM scaffolds with different ECM concentrations on BMSC proliferation. For the CCK-8 assay, we first placed a 3.5 mm diameter circular scaffold into a 96-well plate and separately seeded 5000 cells on the scaffold of each well. Then, 100 μl DMEM/F12 medium was separately added to each well after the cells were attached to the wall. After 1, 4 and 7 days of culture, 100 μl of working solution (CCK-8 reagent/cell culture medium = 1:9 volume ratio) was added to each well and incubated for 2 hours at 37 °C. Then, the liquid in each well was transferred to a new 96-well plate, and the optical density of the CCK-8 working solution at 450 nm was detected by an enzyme marker.

#### Cell attachment and spreading evaluation

BMSC attachment and spreading on the ECM scaffolds were observed by confocal microscopy. Briefly, 1 × 10^6^ BMSCs were seeded onto scaffolds and incubated at 37 °C in 5% CO^2^ for 7 days. Then, the scaffolds were fixed with 4% paraformaldehyde for 30 min and lysed with 0.3% Triton X-100 for 20 min. After that, the samples were blocked with immunoblocking solution for 30 min. Finally, rhodamine-phalloidin and DAPI were used to stain the cytoskeleton and nucleus of the cells on the scaffolds.

### Chondrogenic differentiation assay of pure ECM scaffolds

To evaluate the effects of the three ECM scaffolds with different ECM concentrations on BMSC chondrogenic differentiation, 1 × 10^6^ BMSCs were implanted on the scaffolds. After culturing in complete chondrogenic cultures (Cyagen, China) for 14 days, RT–qPCR was used to detect the expression of cartilage-specific genes. Primer sequences for quantitative RT–PCR are shown in Table S1. Detailed experimental procedures are available in Supplementary File section 1.4.

### Fabrication and release behaviour of the ECM/GDF-5 hybrid scaffold

The optimal concentration of ECM bioink was mixed with GDF-5 and then transferred to a printer for rapid printing as described in “Preparation of pure ECM scaffolds” section. The ECM/GDF-5 scaffold was successfully prepared, and the scaffold was frozen and formed at − 80 °C.

For the GDF-5 release assay, hybrid scaffolds (*n* = 3) were incubated in PBS (containing 0.5% bovine serum albumin), and supernatants were collected from each well at different time points of 0, 1, 4, 7, 8, 14, 21, 28, 35, and 42 days of scaffold incubation and supplemented with an equal volume of fresh PBS. GDF-5 concentrations released at different time points were quantified using a GDF-5 ELISA kit (R&D Systems) according to the manufacturer’s instructions. Finally, the GDF-5 release percentage curve was plotted.

### In vitro and in vivo endogenous cell migration assay

To determine the effect of the hybrid scaffold on BMSC migration, we evaluated it using vertical cell migration (Transwell chamber assay) in vitro. Then, to further explore the ability of hybrid scaffolds to recruit endogenous MSCs in vivo, we established a cartilage defect model in rats and studied the recruitment of endogenous MSCs by simple defects, ECM scaffolds, and ECM/GDF-5 scaffolds. More experimental procedures are available in Supporting File section 1.5.

### Effect of GDF-5 on SMSC chondrogenic differentiation

To investigate the effect of GDF-5 on SMSC chondrogenic differentiation, cartilage pellet culture was performed. Next, the extent of chondrogenesis was assessed by HE, Alcian Blue, Safranin O and type II collagen immunohistochemistry (IHC) staining, and the staining procedure was performed according to the manufacturer’s protocol. Finally, the expression of cartilage-related genes and proteins was detected by RT–qPCR and Western blotting. In addition, to investigate the effect of GDF-5 on the MAPK pathway, the protein expression and intracellular distribution of ERK1/2 and P38 in GDF-5-treated SMSCs were detected by Western blotting and immunofluorescence staining. Detailed experimental procedures are available in Supplementary File section 1.6.

### Effect of hybrid scaffolds on SMSC chondrogenic differentiation

To evaluate the chondrogenic differentiation capacities in different groups, the expression levels of cartilage-specific genes in the cells cultured on different scaffolds were measured at 14 days. Primer sequences for quantitative RT–PCR are shown in Table S1. Detailed experimental procedures are available in Supplementary File section 1.7.

### In vivo animal studies

All animal experiments in this study were approved by the Institutional Animal Care and Use Committee at the PLA General Hospital. Forty skeletally mature New Zealand White rabbits (male, 2.8-3.2 kg, 6 months old) were used as animal models and randomly allocated to 4 groups, with 10 rabbits in each group: (A) the negative control group, (B) the ECM group, (C) the ECM/GDF-5 group and (D) the sham group. After taking the samples, the regenerative cartilage tissue in the defect site among the four groups was compared by macroscopic evaluation and micro-CT analysis. The images were scored according to the International Society for Cartilage Repair (ICRS) macroassessment guidelines (Table S2). Subsequently, histomorphological staining, including H&E, safranin-O/fast green, Sirius red, and collagen II immunohistochemical staining, was performed to evaluate the histological properties of regenerative cartilage. All staining images were scored according to the O’Driscoll scoring system evaluation guidelines (Table S3). In addition, the mechanical properties of the samples were tested with a biomechanical testing machine (Bose, 5100). After that, the total glycosaminoglycan (GAG) and collagen contents in the regenerative tissue were examined by a GAG content DMMB colorimetric kit and a hydroxyproline assay kit according to the manufacturer’s instructions. Detailed experimental procedures are available in Supplementary File sections 1.8, 1.9, 1.10, 1.11 and 1.12.

### Statistical analysis

All data were analysed by using GraphPad Prism 8.0 statistical software, and differences between groups were compared with one-way ANOVA and Student’s t test. All quantitative data are shown as the means ± standard deviations (SDs), with a statistical significance of *p* < 0.05 expressed between groups.

## Results

### Preparation and characterization of ECM bioink

#### Gelation status of ECM at different concentrations

**Fig. 2:**
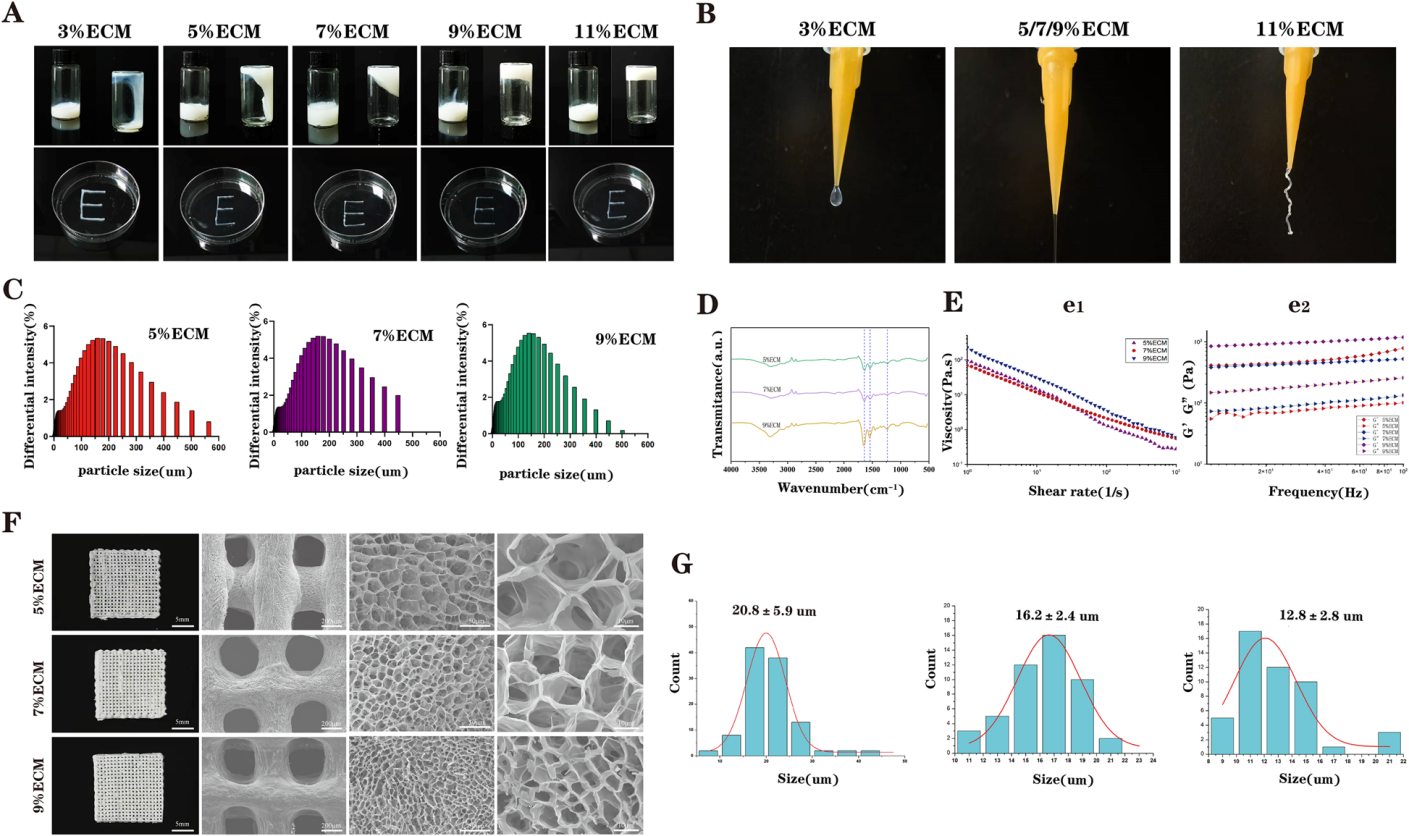
Preparation and characterization of the ECM bioinks and scaffolds with different ECM concentrations. **A** and **B** Gelation status of ECM bioinks at five concentrations; **C** Particle size distributions of 5, 7 and 9% ECM bioinks; **D** FTIR spectra of ECM bioinks; **E** Rheological characterization of ECM bioinks; **F** Macroscopic and SEM images of 5, 7 and 9% pure ECM scaffolds; **G** Pore size distributions of ECM scaffolds

The gelation state of bioinks is crucial for subsequent scaffold construction. As shown in Fig. 2(A-B), the gel of 3% ECM was droplet-like at the nozzle tip and could not retain the shape of the print fibres. The overgelation of 11% ECM resulted in the poor shape of the printed fibres and scaffolds. In contrast, the 5/7/9% ECM showed good gelation conditions, and the printed fibres were smooth, so we chose these three concentrations for further investigation.

#### Particle size distribution

The particle size distributions of ECM materials treated with three different concentrations (5/7/9%) are shown in Fig. 2C. In detail, the particle size distributions of 5% ECM ranged from 3 μm to 448 μm (mean: 133 μm). The 7% ECM ranged from 3 μm to 448 μm (mean: 125 μm), and the 9% ECM ranged from 3 μm to 448 μm (mean: 140 μm). Compared to the diameter of the print nozzle (500 μm), the size of the particles is much smaller, which is one of the basic conditions for printing with ECM bioinks.

#### FTIR spectroscopy

We used FTIR spectroscopy to characterize the structural changes of the ECM at different concentrations. As depicted in Fig. 2D, there were no significant differences among the characteristic peaks of the amine group (C − N stretching vibration and N − H bending vibration of an aliphatic secondary amine) at 1580 cm^− 1^ (blue arrow), the peak of the carbonyl group at 1732 cm^− 1^ (red arrow) and a peak at 3348 cm^− 1^ (a characteristic peak of the N − H stretching vibration of an aliphatic secondary amine, black arrow) for the 5, 7, and 9% ECM, indicating that they all retained the basic components of the natural chondrocyte ECM.

#### Rheological characterization

To further study the printability of bioinks, rheological characterization of the ECM bioinks with different concentrations was performed. As shown in Fig. 2E (e2), the storage modulus (G′) and loss modulus (G″) of the 5, 7, and 9% ECM bioinks maintained good stability under increasing angular frequency. Furthermore, the shear rate-dependent viscosity is crucial for the printability of the bioink. As shown in Fig. 2F (e1), with increasing shear rate, all viscosities of the three ECM bioinks were obviously decreased, indicating that they all have excellent shear-thinning properties.

### Characterization of ECM scaffolds

As shown in Fig. 2F, the milky ECM scaffolds were uniform in terms of wire diameter and pore size. Then, the microstructures of the three scaffolds were observed by using SEM, and all the scaffolds formed hierarchical macro/microporous structures. In addition, it can be seen that with increasing ECM concentration, the diameter of interconnected micropores on the scaffold fibres gradually decreased. As shown in Fig. 2F, the pore size distributions of the three scaffolds (5, 7, and 9%) were also relatively uniform, and their pore sizes were 20.8 ± 5.9 μm, 16.2 ± 2.4 μm and 12.8 ± 2.8 μm, respectively.

Subsequently, based on the linear region slope of 0 − 10% strain in the stress–strain curve, the Young modulus of the three scaffolds was calculated. The results suggested that the Young moduli of the 5, 7, and 9% ECM scaffolds were 63.7 ± 12.58 KPa, 97.0 ± 11.1 KPa and 224.6 ± 19.2 KPa, respectively (Fig. S1A), which also had a certain gap with the mechanical strength of natural AC. Finally, the porosities of the three scaffolds were 81.6, 73.8 and 65.9%, as shown in Fig. S1B.

### Cytocompatibility of ECM scaffolds

#### BMSC identification

Third-generation BMSCs were used to identify phenotypic characteristics. The flow cytometry results of BMSCs (Fig. S2) showed that BMSCs expressed the cell surface markers CD105 (99.6%) and CD90 (99.9%) but not CD34 (0.16%) or CD45 (02.88%), demonstrating that these BMSCs were a pure MSC population with no haematopoietic or endothelial cells.

#### Cell viability and proliferation

**Fig. 3:**
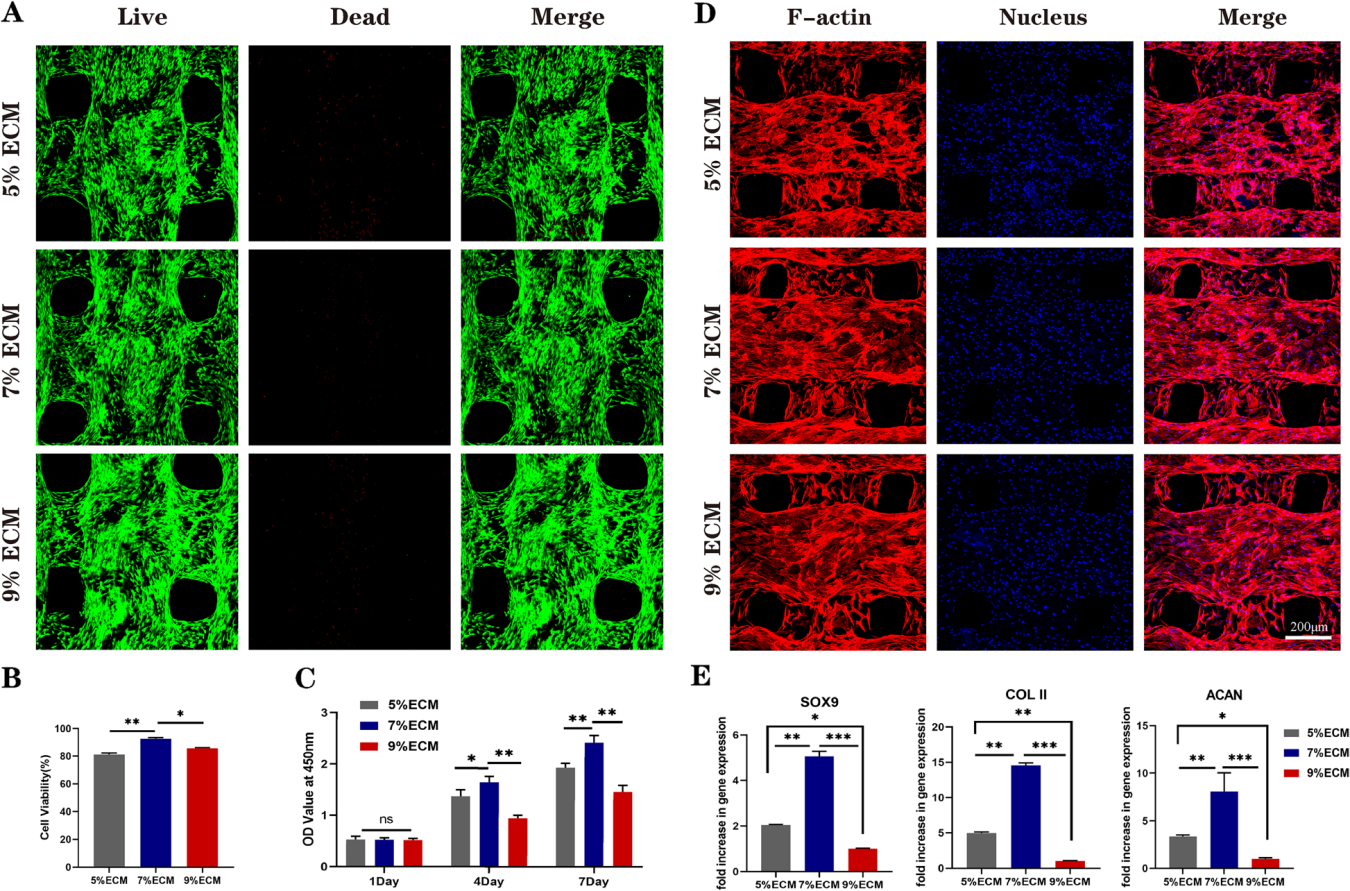
Biocompatibility and chondrogenic differentiation analysis of pure ECM scaffolds. **A** Live/dead staining (green: live cells, red: dead cells) of BMSCs on the pure ECM scaffold; **B** Percentage of live cells on scaffolds (*n* = 3); **C** CCK-8 assay results of BMSCs cultured on ECM scaffolds for 1 day, 4 days, and 7 days (*n* = 4); **D** Morphology of BMSCs on the pure ECM scaffold (red: F-actin, blue: nucleus); **E** Expression of SOX 9, ACAN and Col 2A1 of the SMSCs on 5, 7 and 9% ECM scaffolds (*n* = 3). Statistical analysis: *p < 0.05, **p < 0.01, ***p < 0.001

BMSCs were cultured on the ECM scaffolds with three different ECM concentrations for 7 days, and then the viability of the cells on the scaffolds was observed by live/dead staining. The confocal microscopy results (Fig. 3A) showed that the majority of BMSCs were live cells (green), and only very few BMSCs were dead cells (red). In addition, further quantitative analysis showed that the proportion of live cells on the scaffolds was the highest when the ECM concentration was 7% (Fig. 3B).

To further screen out the optimal ECM concentration, the CCK-8 assay (Fig. 3C) was used to evaluate cell proliferation after culturing on scaffolds after 1, 4 and 7 days. The results indicated that the OD values increased with time for all three scaffold groups, and at 4 and 7 days, the OD values were highest for the 7% ECM group, which was consistent with the live and dead staining results. In conclusion, 7% ECM may be more conducive to BMSC proliferation.

#### Cell attachment and spreading

The attachment and spreading of BMSCs cultured on the ECM scaffolds after 5 days were evaluated by cytoskeleton staining. As shown in Fig. 3D, the cells on the ECM scaffolds with three different ECM concentrations were uniformly distributed and showed good spindle-shaped morphology, which indicated that all the ECM scaffolds could support cell attachment and spreading well.

### In vitro chondrogenic differentiation analysis of ECM scaffolds

To evaluate the effects of the three ECM scaffolds with different ECM concentrations on BMSC chondrogenic differentiation, RT–qPCR was used to detect the expression of cartilage-specific genes. After chondrogenic induction for 14 days, the expression levels of genes (SOX9, ACAN, COL1 and COL2) in the three ECM groups were recorded and are shown in Fig. 3E. The results suggested that the chondrogenic genes (SOX 9, ACAN and COL 2) of the BMSCs in all the ECM groups were significantly upregulated compared with those of the blank control group, while the expression of COL1 was not significantly different. Furthermore, the ECM scaffold with a 7% ECM concentration had the strongest promoting effect on BMSC chondrogenic differentiation. In conclusion, combined with the biocompatibility assessment results, we selected 7% ECM scaffolds for subsequent experiments.

### Fabrication and characterization of the ECM/GDF-5 scaffold

#### Release behaviour of GDF-5

To improve the recruitment and chondrogenic capability of the ECM scaffolds, GDF-5 was incorporated into 7% ECM bioinks, and then 3D-printed hierarchical porous ECM/GDF-5 scaffolds were finally fabricated by LDM. As shown in Fig. S3, the GDF-5 in the scaffolds showed significant release in the first 10 days, the cumulative release amount reached 60%, and then the release effect slowed down over time, reaching approximately 85% after 1 month.

#### ECM/GDF-5 scaffolds promoted BMSC migration in vitro and in vivo

**Fig. 4:**
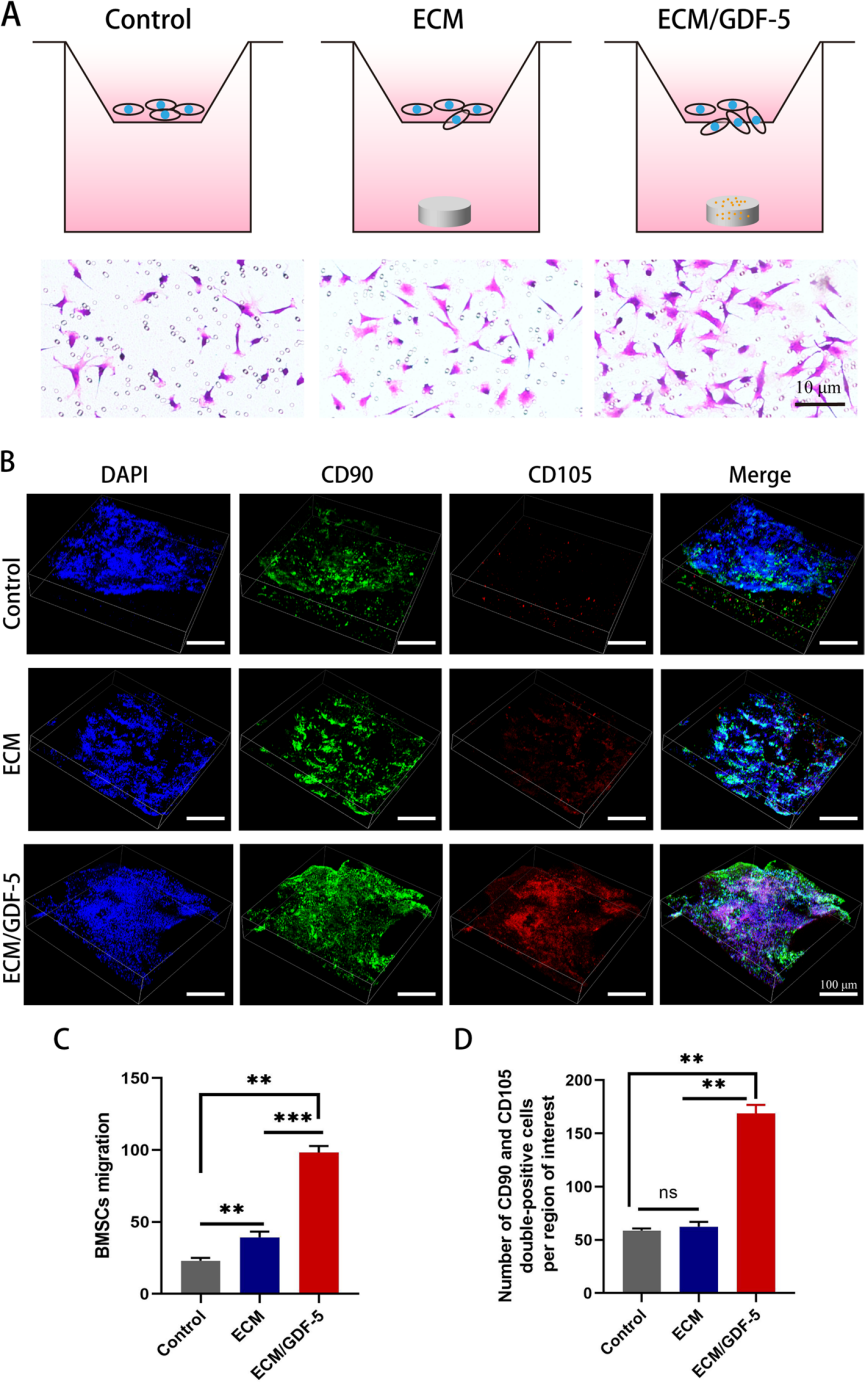
Effects of hybrid scaffolds on BMSC migration in vitro and in vivo. **A** Structure of the Transwell assay and images of crystalline violet staining for the control group, ECM scaffold group and ECM/GDF-5 scaffold group; **B** Confocal images of endogenous MSC migration in the defect area at 2 weeks after surgery; **C** Statistics of the total number of migrating cells in the Transwell assays (*n* = 3); **D** Number of double-positive (CD90 and CD105) MSCs at defect sites in vivo (*n* = 3). Statistical analysis: *p < 0.05, **p < 0.01, ***p < 0.001

To investigate the effects of scaffolds on cell migration in vitro, we used a Transwell chamber assay, and the scaffolds were placed in the lower chamber, as shown in Fig. 4A. The images and statistical analysis of the crystal violet staining (Fig. 4A, C) revealed that the number of migrated BMSCs in the ECM/GDF-5 scaffold group was significantly higher than that in both the ECM scaffold and control groups, indicating that the ECM/GDF-5 scaffold could enhance BMSC migration in vitro. Interestingly, we found that the ECM scaffold group had a certain recruitment capacity compared with the control group, which may be attributed to the retention of some biochemical cues in the ECM.

To confirm that ECM/GDF-5 scaffolds can also promote MSC migration in vivo, immunofluorescence staining was performed on the regenerated cartilage tissues of the three groups 7 days after the implantation of the scaffolds into rabbit AC defect models. The images and statistical analysis of the immunofluorescence staining (Fig. 4B, D) demonstrated that the total number of CD90 and CD105 double-positive cells in the ECM/GDF-5 scaffold group was highest compared with that in the control and ECM scaffold groups, indicating that GDF-5 released from the scaffold could enhance the migration of endogenous MSCs to the defect site and then promote the regeneration of AC in vivo.

### Effect of GDF-5 on BMSC chondrogenic differentiation

#### GDF-5 promoted BMSC chondrogenic differentiation

**Fig. 5:**
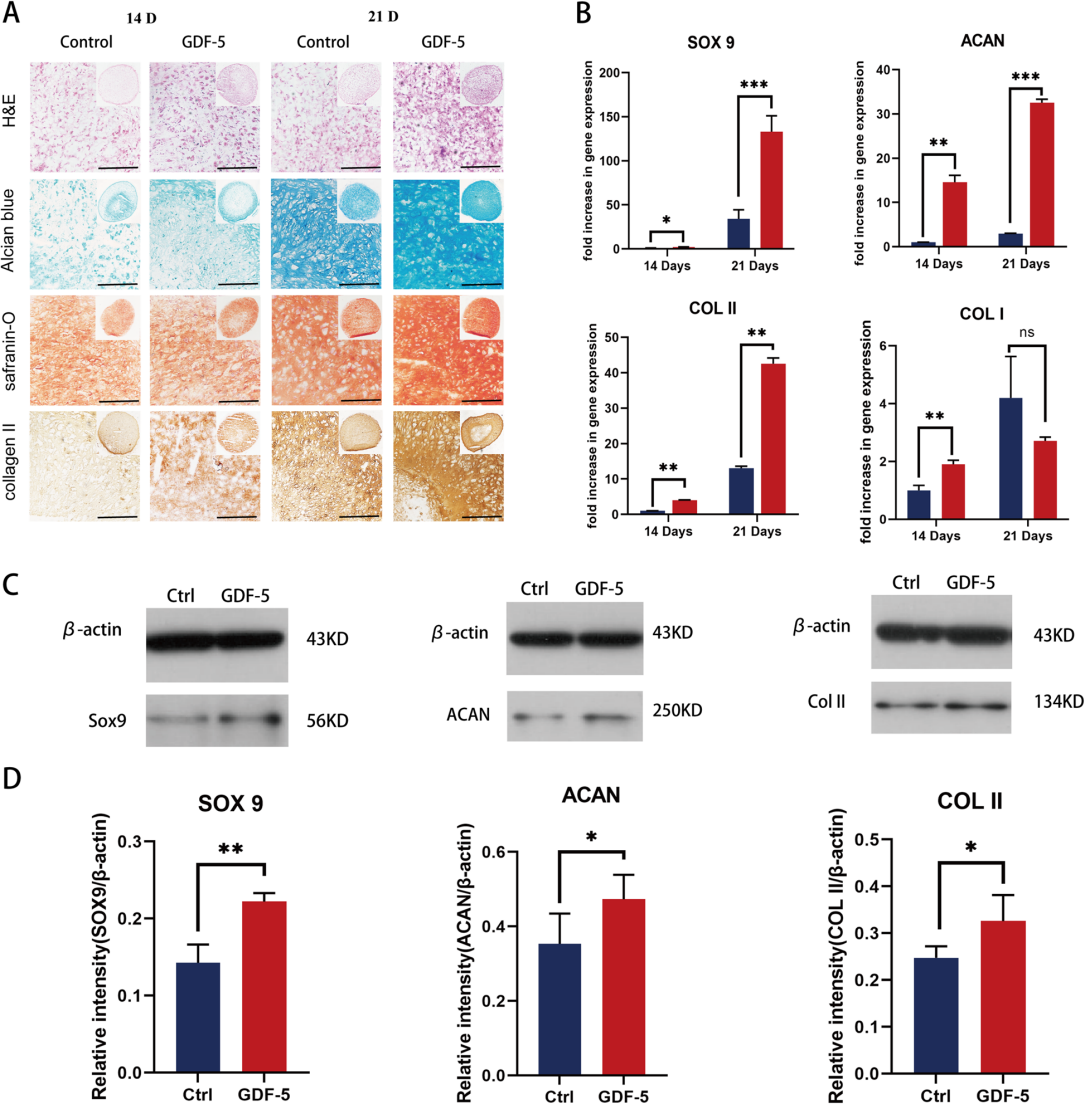
Effect of GDF-5 on BMSC chondrogenic differentiation in vitro. **A** H&E, Alcian blue, safranin-O staining and collagen II immunohistochemistry of BMSC pellets that were cocultured with or without GDF-5 medium for 14 and 21 days. Scale bars are 100 μm. **B** Expression of SOX 9 ACAN, Col2A1, and Col 1 in BMSC pellets (*n* = 3); **C** Western blot detection of chondrogenic markers SOX9, ACAN and COLII at 14 days (*n* = 3); **D** Expression levels of SOX 9, Aggrecan, COL II in quantitative analysis. Statistical analysis: *p < 0.05, **p < 0.01, ***p < 0.001

A cartilage pellet culture system was used to investigate the effects of GDF-5 on BMSC chondrogenic differentiation. As shown in Fig. 5A, histological and immunohistochemical staining were used to evaluate chondrogenic differentiation in the control and experimental groups after 14 and 21 days of chondrogenic induction. The images of H&E staining (Fig. 5A) suggested that the Gdf-5 group had better cell numbers and ECM production at both time points than the control group. In addition, the safranin O and Alcian blue staining results (Fig. 5A) revealed that GDF-5 could significantly enhance proteoglycan production. Furthermore, the IHC staining of collagen II (Fig. 5A) showed that the staining of the GDF-5 group was deeper than that of the control group, indicating that GDF-5 can also promote the deposition of type II collagen in cartilage pellets.

Finally, we further investigated the enhanced effects of GDF-5 on BMSC chondrogenic differentiation at the gene and protein expression levels. As shown in Fig. 5B, the expression of cartilage-specific genes (SOX 9, ACAN and COL 2) in the GDF-5 group was significantly upregulated compared with that in the control group at 14 and 21 days. In addition, we also found that the expression levels of COL1 were not significantly different between the GDF-5 group and the control group at 21 days. Furthermore, the Western blot and statistical analysis results (Fig. 5C, D) demonstrated that the expression levels of cartilage-related proteins, including SOX 9, ACAN and COL 2, were significantly upregulated after GDF-5 treatment, which was consistent with the PCR results. In conclusion, the above results suggested that GDF-5 could significantly promote BMSC chondrogenic differentiation.

#### GDF-5 affected BMSC chondrogenic differentiation by upregulating the phosphorylation levels of Erk 1/2 and P38

**Fig. 6:**
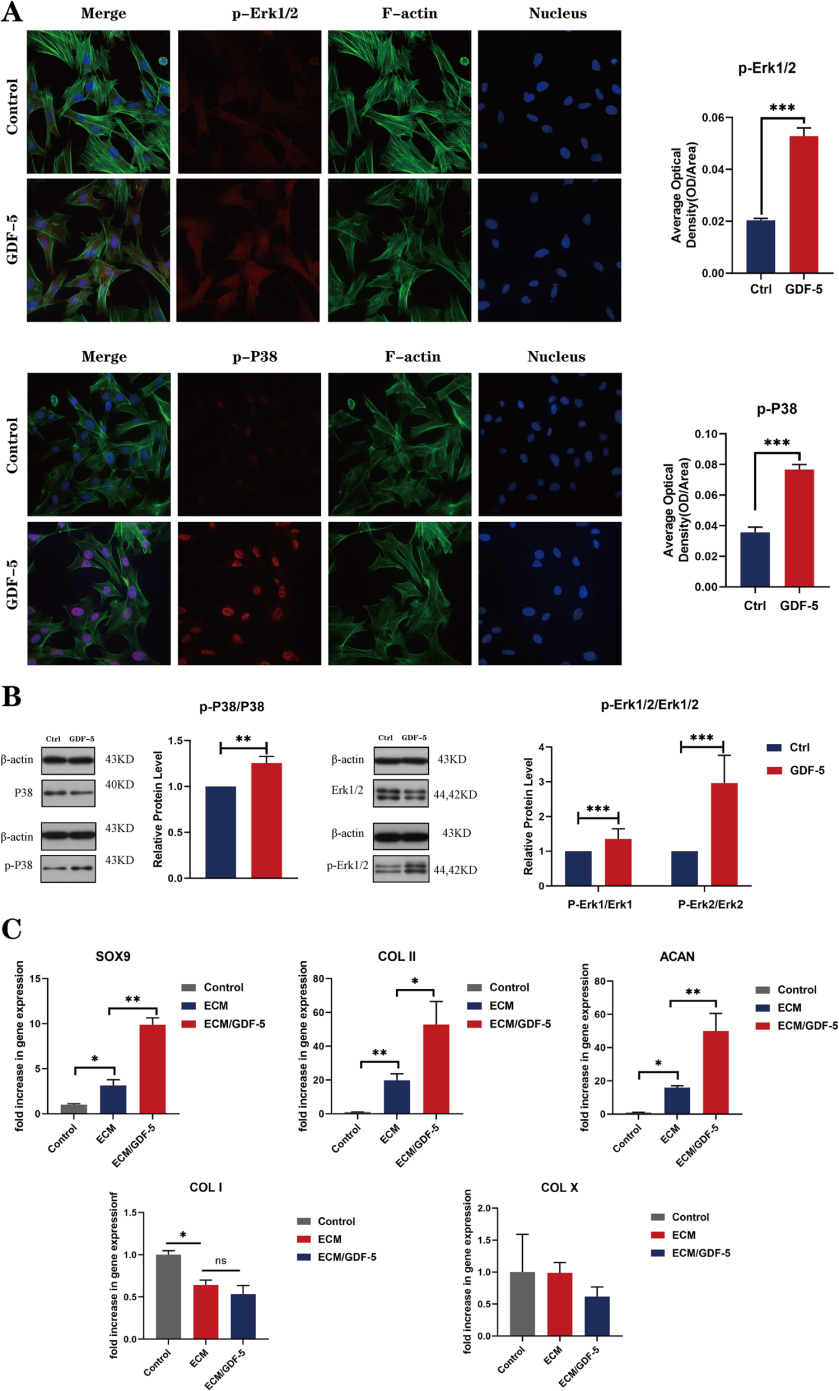
GDF-5 promoted BMSC chondrogenic differentiation by upregulating the phosphorylation of Erk1/2 and P38. **A** Immunofluorescence detection of p-Erk1/2 and p-P38. (F-actin: green, nucleus: blue, and protein: red). Scale bars are 25 μm. Quantitative analysis of the average optical density of fluorescence images. Data are presented as the means ± SDs (*n* = 3); **B** WB analysis of the expression levels of Erk 1/2, p-Erk 1/2 and p-P38 and quantitative analysis of the expression levels of Smad 2/3, p-Smad2 and p-Smad3. Data are presented as the means ± SDs (*n* = 3); **C** Statistics of the total number of migrating cells in the Transwell assays (*n* = 3); **D** Expression of SOX 9, ACAN, COL II, COL I and COL X in SMSCs on hybrid ECM scaffolds (*n* = 3). Statistical analysis: *p < 0.05, **p < 0.01, ***p < 0.001

Previous studies have shown that GDF-5 can promote the chondrogenic differentiation of the chondrogenic cell line ATDC5 by enhancing the phosphorylation of P38. As one of the MAPK families, P38 together with Erk plays a key role in the process of chondrogenesis and plays a nonnegligible role in the chondrogenic differentiation of MSCs. To elucidate the biological mechanism of GDF-5 on BMSC chondrogenic differentiation, we used immunofluorescence staining and Western blot analysis to detect the phosphorylation levels of Erk 1/2 and P38 in cells treated with GDF-5. As shown in Fig. 6A, the immunofluorescence staining results showed that the fluorescence intensities of p-Erk 1/2 and p-P38 in the GDF-5-treated group were significantly increased. Subsequently, Western blot and semiquantitative analysis (Fig. 6B) also demonstrated that the phosphorylation levels of Erk 1/2 and P38 were significantly upregulated in BMSCs exposed to GDF-5. Therefore, we confirmed that GDF-5 enhanced BMSC chondrogenic differentiation by upregulating the phosphorylation levels of Erk 1/2 and P38.

### In vitro chondrogenic differentiation of BMSCs on the ECM/GDF-5 scaffolds

**Fig. 7:**
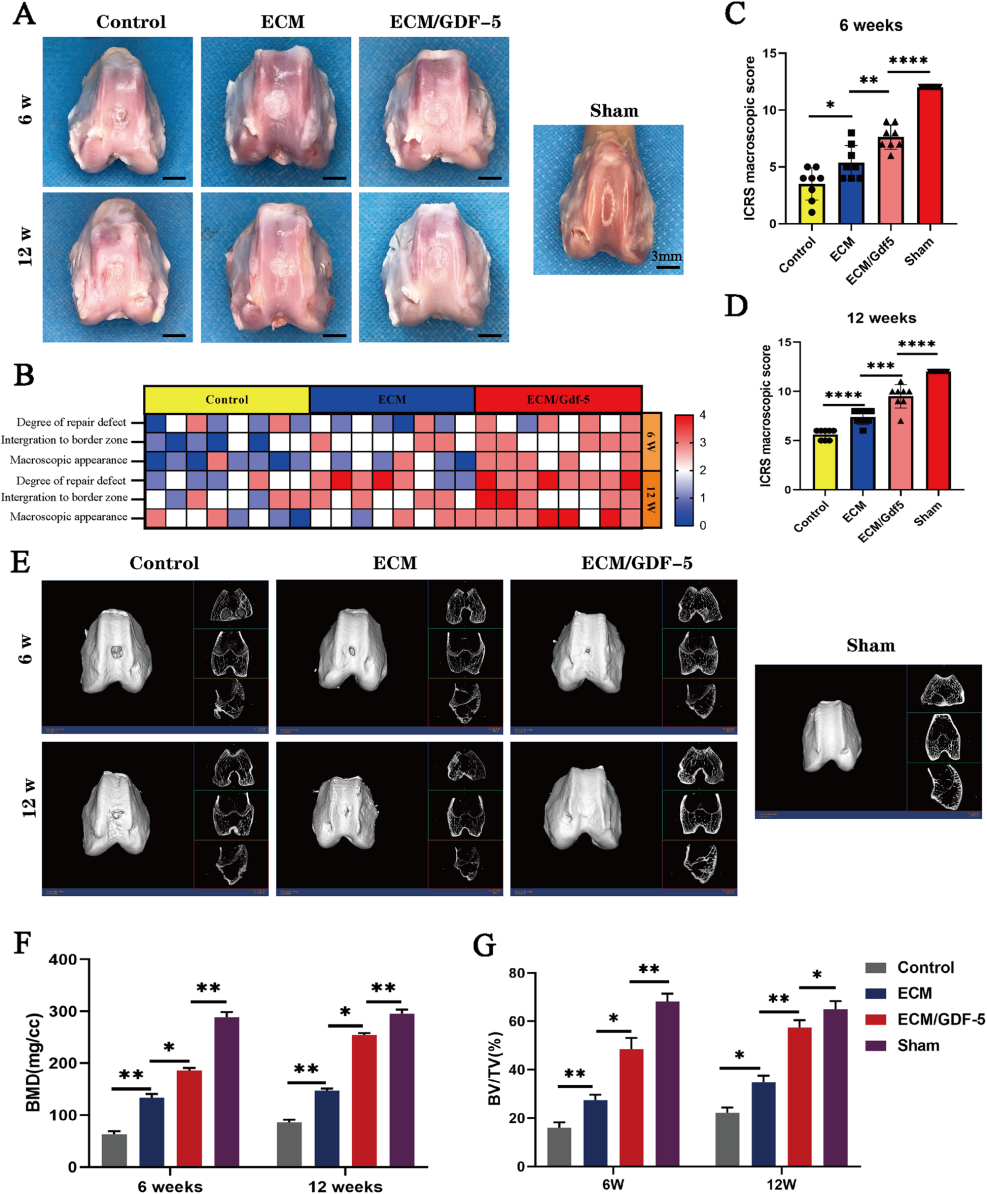
Macroscopic and micro-CT evaluation of repaired knees. **A** Representative macroscopic analysis of repaired tissues at 6 and 12 weeks post-operation; **B** Heatmap of variables for the ICRS scoring; **C** and **D** ICRS scoring for the macroscopic assessment at 6 weeks and 12 weeks (*n* = 8); **E** Micro-CT images of the 2D and 3D reconstruction of repaired cartilage at 6 and 12 weeks post-operation. **F** and **G** Quantitative analysis of BMD and BV/TV in the regenerated area. Statistical analysis: *p < 0.05, **p < 0.01, ***p < 0.001

To investigate the capacity of the ECM/GDF-5 scaffold for BMSC chondrogenic differentiation compared with that of the ECM scaffold, the expression of cartilage-specific genes (SOX9, ACAN and COL II), COL I and COL X was detected by RT–qPCR. After chondrogenic induction for 14 days, the expression levels of genes in the three groups were recorded and are shown in Fig. 7C. The results suggested that the chondrogenic genes (SOX 9, ACAN and COL II) of BMSCs in the ECM/GDF-5 group were significantly upregulated compared with those in the ECM and tissue culture plate (TCP) groups, and the expression of collagen I and collagen X was low in the ECM-based scaffold groups compared with the control group, indicating that GDF-5 in the scaffolds retained the activity and greatly improved the chondrogenic differentiation of cells seeded on the scaffolds.

### In vivo cartilage repair studies

#### Macroscopic and micro-CT evaluation

To fully investigate the role of the ECM and ECM/GDF-5 scaffolds in the promotion of AC regeneration, we implanted them into a rabbit full-thickness AC defect model, and then the regenerative effects were evaluated at 6 and 12 weeks after surgery. As shown in Fig. 7A, at 6 weeks, the cartilage defects in the control group were still evident, while the defects in the ECM group were filled with regenerated tissue, but most of this tissue was white inflammatory granulation tissue, which was significantly different from the surrounding normal cartilage tissue. In contrast, the regenerated cartilage in the ECM/GDF-5 group was significantly more similar to the surrounding normal cartilage and better integrated. At 12 weeks after implantation, although the defect area in the control group was filled with regenerated tissue, it was obviously inflammatory tissue compared with the surrounding normal cartilage. In addition, the inflammatory tissue in the ECM group remained at 12 weeks, while the ECM/GDF-5 group had the best regeneration effects and was the most similar to the normal cartilage tissue in the sham group. Consistent with the gross observation, the International Cartilage Repair Society (ICRS) scores also showed that cartilage regeneration in the ECM/GDF-5 group was significantly better than that in the other two groups at 6 and 12 weeks (Fig. 7B, C, D).

Micro-CT analysis was performed to compare the regenerative cartilage tissue in the defect site among the three groups. The femur samples at two time points were reconstructed in three dimensions, and the two-dimensional images of each section were captured to observe the morphology of the regenerated tissue in the defect area. In addition, bone mineral density (BMD) and bone volume/tissue volume (BV/TV) were used to evaluate subchondral bone. The images of reconstruction (Fig. 7E) showed that the ECM/GDF-5 group had the best regeneration effect compared with the subchondral bone of the control group and the ECM group at two time points. The values of bone mineral density (BMD) and bone volume/tissue volume (BV/TV) in the ECM/GDF-5 group were also better than those in the other two groups at both 6 weeks and 12 weeks after the operation (Fig. 7F, G). In conclusion, the macroscopic and micro-CT evaluation results revealed that the ECM/GDF-5 scaffolds could significantly enhance AC regeneration at both 6 weeks and 12 weeks.

#### Histological evaluation

**Fig. 8:**
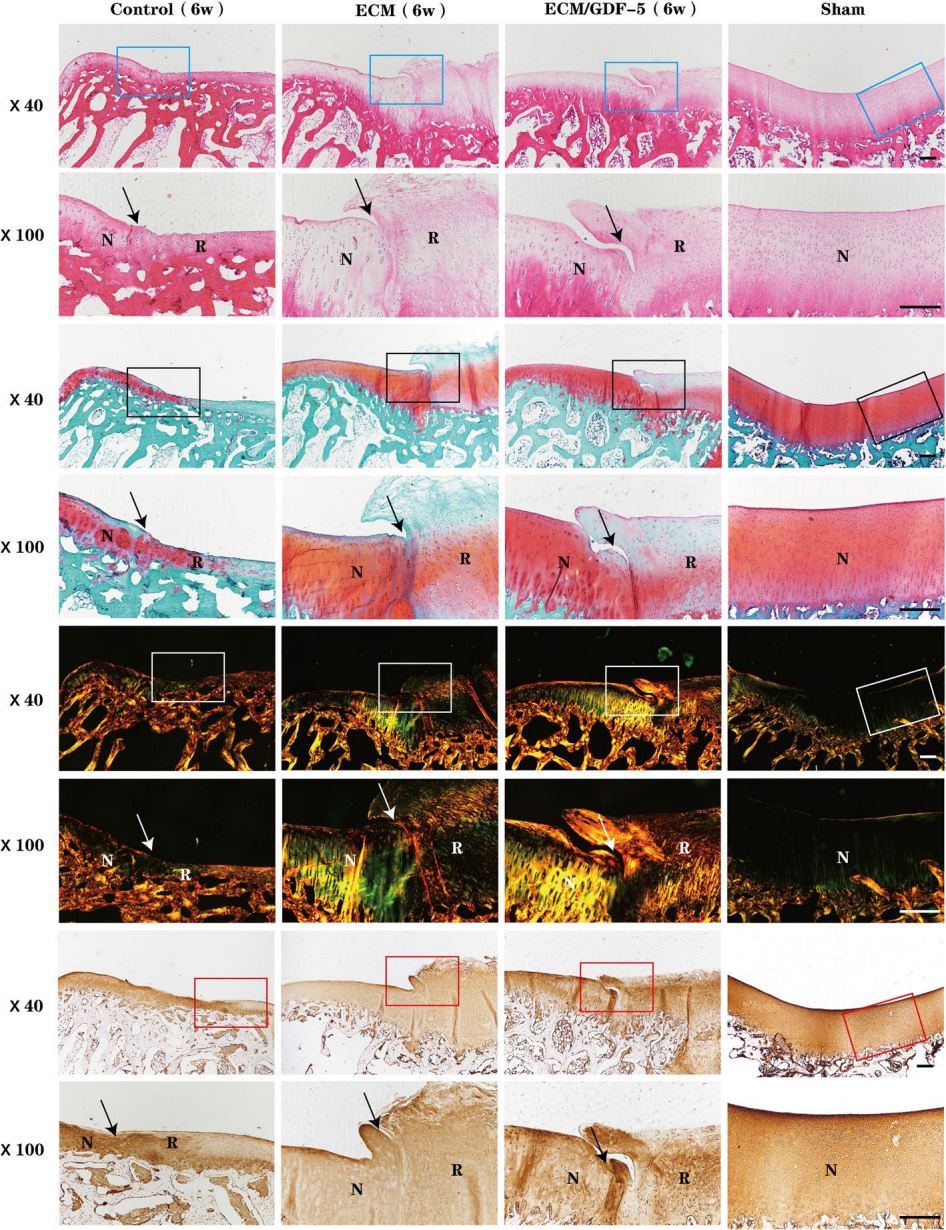
Histomorphological evaluation of repaired tissue at 6 weeks. Histological (H&E, safranin O, and Sirius red staining) and immunohistochemical staining (collagen II) of the defect area. Solid arrows indicate the repair interface. (N: normal cartilage; R: repaired cartilage). Scale bars are 500 μm

The histological properties of the regenerative cartilage were evaluated by histomorphological staining, including H&E, safranin-O/fast green, Sirius red, and collagen II immunohistochemical staining. At 6 weeks, the H&E and safranin-O/fast green staining results (Fig. 8) showed that in the control group, the defect area still had obvious collapse, the regenerated tissue was not typical hyaline cartilage, and the cell arrangement was also disorganized. In the ECM group, although cartilage tissue was regenerated, the surface was covered with flocculent fibrous tissue, and the staining was light. In contrast, in the ECM/GDF-5 group, although the integration of the regenerated tissue with the surrounding cartilage was poor, the repaired tissue was smoother, and the cell arrangement was more orderly than those in both the control and ECM groups. Moreover, the Sirius red and collagen II immunohistochemical staining results (Fig. 8) also revealed that the collagen content of the ECM/GDF-5 group was more abundant and that the arrangement was closer to that of the sham group compared with the other two groups.

**Fig. 9:**
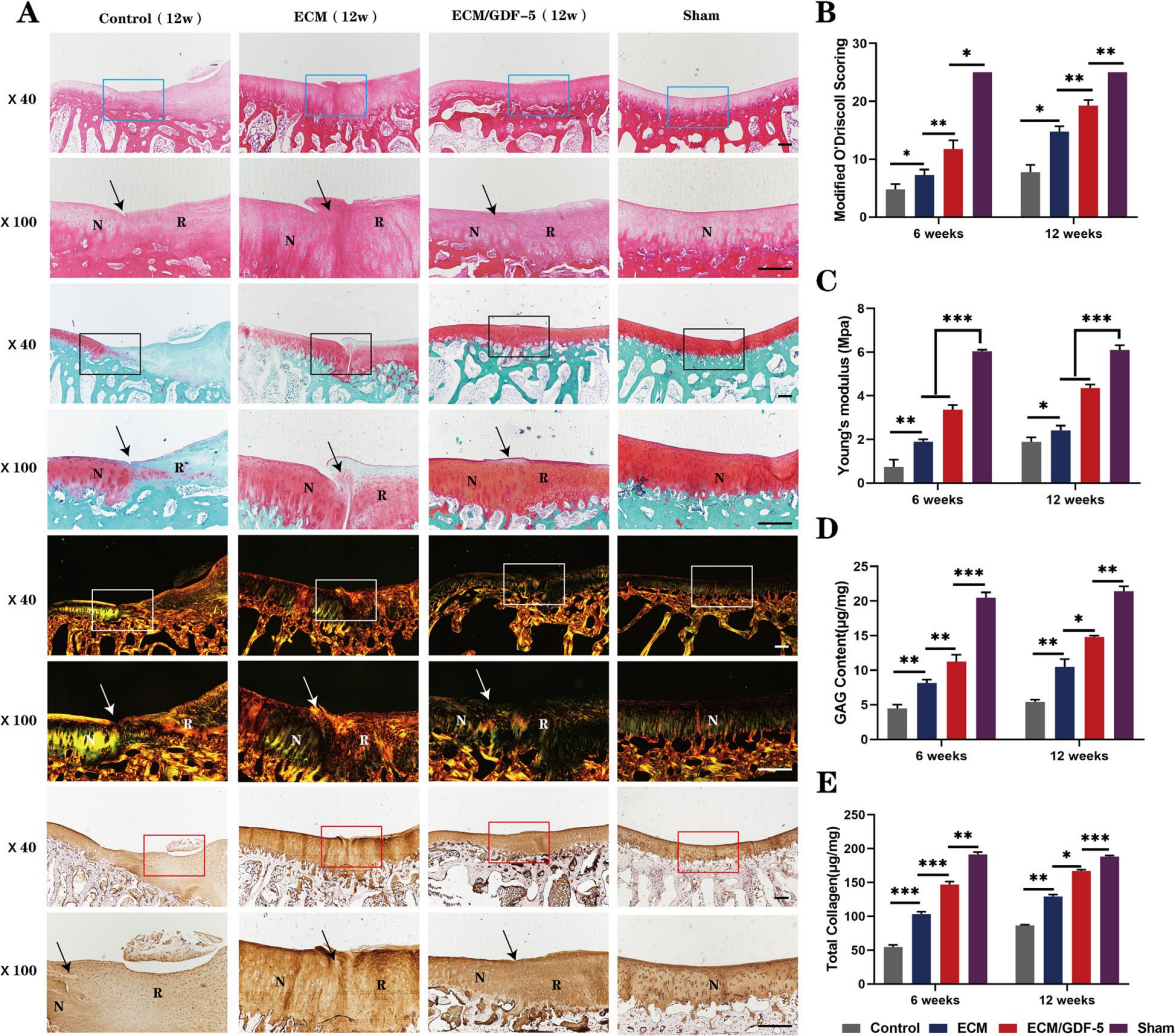
Histomorphological evaluation of repaired tissue at 12 weeks. **A** Histological (H&E, safranin O, and Sirius red staining) and immunohistochemical staining (collagen II) of the defect area. Solid arrows indicate the repair interface. (N: normal cartilage; R: repaired cartilage). Scale bars are 500 μm. **B** Modified O’Driscoll scores for the histological evaluation of cartilage repair after 6 weeks and 12 weeks. Data are presented as the means ± SDs (*n* = 3). **C** Young’s modulus of repaired cartilage in different groups (*n* = 3). **E** and **F** Biochemical assay results showing the GAG and total collagen contents. Statistical analysis: *p < 0.05, **p < 0.01, ***p < 0.001

At 12 weeks (Fig. 9A), the defects in the control group were filled with a large amount of fibrous tissue, and the overall repair effect was the worst. In the ECM group, the repaired tissue was significantly smoother than that at 6 weeks, but there was still more inflammatory tissue. In the ECM/GDF-5 group, the regenerated area was well integrated with the surrounding normal cartilage, and its cell arrangement and collagen content and arrangement were closer to those of the normal cartilage in the sham group. As shown in Fig. 9B, the modified O’Driscoll scoring results were consistent with the histomorphological staining results, indicating that the scores of the ECM/GDF-5 group were higher than those of the control and ECM groups at both 6 weeks and 12 weeks, while there was still a certain difference from the scores of the sham group. In addition, the biochemical analysis results (Fig. 9D, E) also suggested that both the GAG and total collagen content in the ECM/GDF-5 group was highest among all groups (except the sham group) at both time points. In summary, the ECM/GDF-5 scaffold had the strongest effect on the regeneration of AC defects from the perspective of histological evaluation.

#### Biomechanical evaluation

The mechanical properties of the regenerated cartilage were evaluated by biomechanical tests. The testing results (Fig. 9C) showed that the Young’s modulus of the regenerated cartilage in the ECM/GDF-5 group was significantly higher than that in both the control and ECM groups at both time points, indicating that the mechanical properties of regenerated tissue in the ECM/GDF-5 group were best. However, the mechanical properties of all three groups (control, ECM and ECM/GDF-5 groups) were significantly different from those of the normal cartilage of the sham group.

## Discussion

The regeneration of AC has been one of the most important but unsolved challenges in regenerative medicine, and many studies have been devoted to exploring an ideal AC regeneration model [28, 29]. In recent years, in situ AC tissue engineering techniques with endogenous stem cells have made good progress [4, 30]. For in situ tissue engineering, the necessary elements are the regenerated microenvironment and the number of stem cells in the cartilage defect area [7, 31]. A large number of previous studies have shown that decellularized ECM can provide a favourable microenvironment for cell regeneration compared with other biomaterials, which is quite beneficial for the repair of joint defects [32–34]. In traditional tissue engineering, ECM is usually made into lyophilized scaffolds, but its morphology and porosity are very uneven, which cannot provide good growth space for cells [10]. LDM provides a possibility for manufacturing more precise scaffolds by ECM. Compared with the 3D printing of high-temperature melting, LDM can preserve ECM material properties and biological activities to a large extent, which is crucial for maintaining scaffold biocompatibility [15, 16].

In our study, for the first time, a cartilage scaffold with a graded porous structure was prepared by using pure ECM as a bioink in LDM 3D printing. Through experiments, we found that with the increase in ECM concentration in the solvent, the bioink gradually became overgelated, and the most suitable bioinks for printing had 5, 7 and 9% ECM. After the scaffolds were printed by using 5, 7 and 9% ECM bioinks, we found that all the scaffolds had hierarchical porous structures, which could not only promote the exchange of nutrients and oxygen but also provide attachment and adsorption for MSCs and bioactive molecules. In addition, the pore sizes of the three scaffolds decreased gradually with increasing ECM concentration. Previous studies have revealed that the appropriate pore size and porosity have a significant influence on the biological function of cells [35, 36]. Therefore, we investigated the effects of the three scaffolds on the biological function of BMSCs. The proliferation and chondrogenic differentiation results suggested that the 7% ECM scaffolds provide an optimal microenvironment for BMSC cartilage regeneration.

On the other hand, we need to further improve the biochemical cues of scaffolds and the driving force of MSCs to enhance cartilage regeneration. GDF-5, a member of the TGF-β/BMP superfamily, plays a critical role in AC development and regeneration [21]. In particular, in a recent study, Sun et al. found that exogenous GDF-5 enhanced BMSC chondrogenic differentiation and migration in vitro [24]. Therefore, GDF-5 and ECM were mixed for hybrid printing, and LDM 3D printing significantly protected the activity of GDF-5. First, we confirmed the effect of GDF-5 on BMSC chondrogenic differentiation and further explored its biological mechanism based on previous studies. Next, we investigated the effect of the hybrid scaffold on BMSC migration in vivo and chondrogenic differentiation in vitro. The hybrid scaffold showed significantly enhanced migration and chondrogenic differentiation. In conclusion, we found that the ECM scaffold incorporating GDF-5 can not only provide the necessary microenvironment for cellular defect repair but also recruit more stem cells to the defect area in vivo, which has positive significance for in situ AC repair.

To confirm the effects of the scaffolds on AC repair in situ, we implanted them into articular cartilage defects in rabbits. The postoperative results showed that cartilage regeneration in the ECM/GDF-5 group was significantly better than that in the other two groups (control and ECM groups), and the gap between the ECM/GDF-5 group and normal cartilage in the sham group was minimal. Combining the in vitro and in vivo experimental results, we propose a possible mechanism by which the ECM/GDF-5 hybrid scaffold enhances AC regeneration. GDF-5 in the scaffold was released in large quantities in the early stage, which acted on MSCs in the joint cavity, especially BMSCs released after microfracture, causing them to migrate to the defect area. Then, the stem cells adhered to the hierarchical porous structure and finally differentiated into chondrocytes. Specifically, as the main component of scaffolds, decellularized ECM combined with GDF-5 can provide a good regenerative microenvironment for cell proliferation and differentiation. Finally, during the interaction between the cells and the scaffold, along with the degradation of the scaffold, regenerative cartilage was gradually produced and finally filled the defect area.

Although the above results showed that the ECM/GDF-5 hybrid scaffold constructed by LDM 3D printing significantly improved the quality of AC repair in situ, there are still some limitations that need to be further explored. First, due to its low cost and easy availability, we used ECM made from porcine AC. The biosafety of this heterogeneous ECM in clinical application may need to be further verified. Second, the mechanical properties of scaffolds made of ECM alone are poor. How to improve the mechanical properties without affecting their biocompatibility is the focus of our next work. Finally, we need to study the long-term effects before clinical translation in large mammals, such as sheep. Despite the above limitations, this ECM/GDF-5 scaffold printed with LDM technology still has broad prospects in the field of AC in situ tissue engineering and clinical transformation.

## Conclusions

In summary, for the first time, we used ECM alone as a bioink in LDM 3D printing and then successfully fabricated a hierarchical porous ECM scaffold incorporating GDF-5. According to the in vitro and in vivo experimental results, the ECM/GDF-5 scaffolds not only recruited a large number of stem cells to the defect area but also provided an ideal regenerative microenvironment for the MSCs. Finally, animal models have demonstrated that the ECM/GDF-5 scaffolds promote in situ AC repair in vivo, indicating that this scaffold has significant potential for AC in situ regeneration tissue engineering in the future.

## Supplementary Information

**Additional file 1: Supplementary Materials and Methods.** **Fig. S1.** (A) Mechanical properties of 5, 7 and 9% ECM scaffolds. (B) Porosity of 5, 7 and 9% ECM scaffolds. **Fig. S2.** Flow cytometric analysis of MSC-specific surface markers for CD 34, CD 45, CD 90 and CD 105. **Fig. S3.** Release behaviour of ECM/GDF-5 scaffold. **Fig. S4.** Macroscopic and SEM images of FDM-PCL, LDM-PCL and LDM-ECM scaffolds. **Fig. S5. **Biocompatibility and chondrogenic differentiation analysis of the three scaffolds. (A) Live/dead staining (green: live cells, red: dead cells) of BMSCs on the scaffolds. (B) CCK-8 assay results of BMSCs cultured on the scaffolds for 1 day, 4 days, and 7 days (n = 4). (C) Expression of SOX 9, ACAN and Col 2A1 in the SMSCs on three scaffolds (n = 3). Statistical analysis: *p < 0.05, **p < 0.01, ***p < 0.001. **Table S1.** Primer sequences for quantitative RT–PCR. **Table S2.** International Cartilage Repair Society (ICRS) macroscopic evaluation guidelines. **Table S3.** Modified O’Driscoll score system.

## Data Availability

Not applicable.

## Abbreviations

AC: Articular cartilage
LDM: Low-temperature deposition manufacturing
BMSC: Bone marrow mesenchymal stem cell
OA: Osteoarthritis
MF: Microfracture
ACI: Autologous chondrocyte implantation
ECM: Extracellular matrix
MSC: Mesenchymal stem cell
FDM: Fused deposition modelling
WPU: Waterborne polyurethane
FTIR: Fourier transform infrared
SEM: Scanning electron microscopy
SDs: Standard deviations
TCP: Tissue culture plate
IHC: Immunohistochemistry
ICRS: International Cartilage Repair Society
BMD: Bone mineral density
BV/TV: Bone volume/Tissue volume
