# Supplementary material for: Hierarchical porous ECM scaffolds incorporating GDF-5 fabricated by cryogenic 3D printing to promote articular cartilage regeneration
Source: Biomater Res. 2023 Feb 5;27:7. doi: 10.1186/s40824-023-00349-y (PMC9899401; doi:10.1186/s40824-023-00349-y)
Supplement: Supplementary file 1 — Additional file 1: Supplementary Materials and Methods. Fig. S1. (A) Mechanical properties of 5, 7 and 9% ECM scaffolds. (B) Porosity of 5, 7 and 9% ECM scaffolds. Fig. S2. Flow cytometric analysis of MSC-specific surface markers for CD 34, CD 45, CD 90 and CD 105. Fig. S3. Release behaviour of ECM/GDF-5 scaffold. Fig. S4. Macroscopic and SEM images of FDM-PCL, LDM-PCL and LDM-ECM scaffolds. Fig. S5. Biocompatibility and chondrogenic differentiation analysis of the three scaffolds. (A) Live/dead staining (green: live cells, red: dead cells) of BMSCs on the scaffolds. (B) CCK-8 assay results of BMSCs cultured on the scaffolds for 1 day, 4 days, and 7 days (n = 4). (C) Expression of SOX 9, ACAN and Col 2A1 in the SMSCs on three scaffolds (n = 3). Statistical analysis: *p < 0.05, **p < 0.01, ***p < 0.001. Table S1. Primer sequences for quantitative RT–PCR. Table S2. International Cartilage Repair Society (ICRS) macroscopic evaluation guidelines. Table S3. Modified O’Driscoll score system. [file 40824_2023_349_MOESM1_ESM.docx]

**Supplementary materials for**

Hierarchical porous ECM scaffolds incorporating GDF-5 fabricated by cryogenic 3D printing to promote articular cartilage regeneration

Jiang Wu^1,2#^, Liwei Fu^2,3#^, Zineng Yan^1,2#^,Yu Yang^5^, Han Yin^2^, Pinxue Li^2,3^ , Xun Yuan^1,2^, Zhengang Ding^1,2^, Teng Kang^1^, Zhuang Tian^2^, Zhiyao Liao^2,3^, Guangzhao Tian^2,3^, Chao Ning^2^, , Yuguo Li^2^, Xiang Sui^2^, Mingxue Chen^2,4^*, Shuyun Liu^2^*, Quanyi Guo^1,2,3^*

1: Guizhou Medical University, Guiyang 550004, Guizhou Province, People’s Republic of China

2: Institute of Orthopedics, Chinese PLA General Hospital; Beijing Key Laboratory of Regenerative Medicine in Orthopedics; Key Laboratory of Musculoskeletal Trauma & War Injuries PLA; No. 28 Fuxing Road, Haidian District, Beijing 100853, People’s Republic of China

3: School of Medicine, Nankai University, Tianjin 300071, People’s Republic of China

4:Department of Orthopaedic Surgery, Peking University Fourth School of Clinical Medicine, Beijing Jishuitan Hospital, Beijing, 100035, People’s Republic of China

5: Department of Orthopedics, The Second People’s Hospital of Guiyang, 547 Jinyang South Road, Guiyang, Guizhou 550023, China.

#These authors contributed equally to this work

*Corresponding author:

1. Quanyi Guo, Email: [doctorguo_301@163.com](mailto:doctorguo_301@163.com)
2. Shuyun Liu, Email: [clear_ann@163.com](mailto:clear_ann@163.com)
3. Mingxue Chen, Email: chenmingxueplagh@hotmail.com
4. **Supplementary Materials and Methods**
   1. ECM preparation

Briefly, we took the desired articular cartilage from the knee joint of a purchased pig and cut it into evenly sized pieces. The samples were repeatedly frozen for 10 cycles at -80 °C and thawed at room temperature. The samples were homogenized at low temperature (4 °C) and treated with 0.25% trypsin and EDTA solution for 24 h under vigorous stirring. Then, RNA and DNA nucleases (50 U/ml 1DNAseDNAse and 1 U/ml 1RNAse A in 10 mM Tris-HCl, pH 7.5) were treated at 37 °C for 4 h, rinsed with PBSR solution, and finally rinsed with deionized water for 7 d to remove toxic substances and residual reagents.

- 1. Porosity measurement

To determine the porosity of the scaffolds, a classical ethanol replacement method was used in this study. Briefly, the initial volume of ethanol in the measuring cylinder was recorded as V1. Then, the stent was immersed in ethanol for 10 min until the solution was free of air bubbles, at which time the ethanol completely filled the pores of the stent, and the new volume was recorded as V2. After removing the stent, the remaining volume of ethanol was recorded as V3. The porosity was calculated as porosity = (V1 - V3)/(V2 - V3) × 100%. Three parallel replicates were set up for each group.

- 1. Cell culture

In short, the tibia and femur bones of SD rats were separated under sterile conditions. The bone marrow cavity was rinsed repeatedly with PBS containing 0.5% penicillin/streptomycin (Sigma, USA), which was then withdrawn with a syringe. The bone marrow mesenchymal stem cell (BMSC) isolate was centrifuged at 500 × g for 10 min at a concentration of 10% (v/v) FBS and 1% (v/v) penicillin‒streptomycin (GIBCO, Biosciences, Inc., Inc. Ireland) in basal medium (DMEM) and incubated at 37 °C containing 5% CO_2_, with the medium changed every two days. When the cells reached 90% confluence, they were subcultured at a rate of 1:2. BMSCs from the third to fourth passages were used in this experiment.

- 1. Chondrogenic differentiation assay of pure ECM scaffolds

The expression levels of genes related to BMSC chondrogenic differentiation in different scaffold materials were analysed. BMSCs were inoculated on 5%, 7% and 9% ECM scaffolds and cultured in chondrogenic medium for 14 d. Total RNA was isolated from BMSCs using the Cytosolic Total RNA Isolation Kit (Foregene, Chengdu, China). cDNA was synthesized from total RNA using P5 × RT Master Mix (Toyobo, Osaka, Japan). 2× RealStar Green Fast Mix (Genstar, Beijing, China) was used to detect chondrogenic activity. The expression of cartilage-specific genes was examined using 2× RealStar Green Fast Mix (Genstar, Beijing, China). Gene expression was normalized by the ΔΔCt method, and relative gene expression was quantified by the gene expression of the housekeeping gene GAPDH. The target gene (Col 2A1, Sox 9, ACAN)-specific primary primers are shown in Table S1. Three parallel replicates were set up for each group.

- 1. In vitro and in vivo endogenous cell migration assay

A vertical cell migration assay (Transwell assay) was used to evaluate the effects of the scaffolds on BMSC migration in vitro. Briefly, cells (2 × 10^4^) were seeded in the upper chamber of a Transwell system. The lower chamber was filled with 5% FBS as the control group, and the 5% FBS+ECM scaffold and 5% FBS+ECM/GDF-5 scaffold groups were used as the experimental groups. Each group was replicated in 3 wells. After 24 h of incubation, the chamber was removed and fixed with 4% polyformaldehyde for 30 min, and the nonmigrated cells were carefully removed from the upper chamber. After washing with PBS, the Transwell permeating membranes were removed, placed on slides, stained with 0.1% crystal violet solution for 15 min, washed three times with distilled water, and covered with water-based sealant. Images of the migrating BMSCs were observed by light microscopy, and the number of migrating cells was counted in three microscope views per well. The number of migrating BMSCs was calculated using ImageJ software.

To further explore the ability of the composite scaffolds to induce the migration of endogenous mesenchymal stem cells in vivo, we established a cartilage defect model in rats and studied the recruitment of endogenous mesenchymal stem cells by simple defects, ECM scaffolds, and ECM/GDF-5 scaffolds. A total of 9 18-week-old, 200-250 g SD rats were randomly divided into 3 groups. Under sterile conditions, a 2.0 mm diameter trephine was used to create a cartilage defect of approximately 1 mm depth at the femoral trochlea. After implantation of the scaffold, the patella was reduced, and the joint cavity and skin were sutured. The rats were euthanized 2 weeks after surgery, and the new tissue was harvested from the defect area. CD90 and CD105 were defined as the specific markers of MSCs, and the effect of the composite scaffold on the migration of endogenous stem cells was detected by immunofluorescence double staining. Briefly, the removed tissue was fixed with 4% paraformaldehyde for 30 min, washed three times with PBS, and then permeated with Triton X-100 (0.5%) for 30 min. After washing with PBS, the tissue was blocked with immune blocking solution for 30 min. The samples were then incubated with anti-CD90 and anti-CD105 primary antibodies overnight at 4 °C. The following day, unbound primary antibodies were washed with PBS, and the samples were incubated with Alexa Fluor 488- and Fluor 594-conjugated secondary antibodies (Abcam, Cambridge, UK) for 1 h at room temperature. DAPI staining was performed for 15 min. Confocal microscopy was used to observe the number of CD90 and CD105 double-positive stem cells.

- 1. Effect of GDF-5 on SMSC chondrogenic differentiation

To investigate the ability of GDF-5 to enhance BMSC chondrogenic differentiation, as described in previous studies performed by making cells into cell pellet cultures, we added chondrogenic differentiation-inducing medium (Cyagen, China) with and without GDF-5 and continued the culture for 14 and 21 d. The cultured chondrocyte pellets were subjected to frozen sectioning at both time points and then fixed in 95% ethanol. Next, the extent of chondrogenesis was assessed by HE, Alcian Blue, safranin O and type II collagen immunohistochemistry staining, and the staining procedures were performed according to the manufacturer's protocol.

The expression levels of the target genes (Col 2A1, Sox 9, ACAN) of pellets in different groups were analysed. Total RNA was isolated from BMSCs using the Cytosolic Total RNA Isolation Kit (Foregene, Chengdu, China). cDNA was synthesized from total RNA using P5 × RT Master Mix (Toyobo, Osaka, Japan). 2× RealStar Green Fast Mix (Genstar, Beijing, China) was used to detect chondrogenic activity. The expression of cartilage-specific genes was examined using 2× RealStar Green Fast Mix (Genstar, Beijing, China). Gene expression was normalized by the ΔΔCt method, and relative gene expression was quantified by the gene expression of the housekeeping gene GAPDH.

To determine the expression levels of chondrogenesis-related proteins, we used Western blotting to detect the expression of Sox9, collagen II, aggrecan, P38, phospho-P38, Erk1/2, and phospho-Erk1/2 proteins related to cartilage formation. Chondrospheres were treated with chondrogenic medium with or without GDF-5 chondrogenic for 14 and 21 d and used to detect chondrogenic-related proteins. Bone marrow stem cells were inoculated in 6-well plates and treated with GDF-5-containing and GDF-5-free chondrogenic culture medium for 24 h, which were used to detect chondrogenic differentiation-associated pathway proteins. Total proteins from the above samples were collected using the Cellular Protein Extraction Kit. The protein samples and buffers were mixed and heated, and the purified proteins were separated on SDS%-PAGE gels. Finally, the proteins were transferred to polyvinylidene fluoride (PVDF) membranes, and the PVDF membranes were covered with 5% skim milk. The membranes were incubated overnight at 4 °C with anti-β-actin (1:5000, Immunoway, TX, USA), anti-collagen type II (1:1000, Novus, NY, USA), anti-Sox9 (1:1000, Abcam, Cambridge, England), anti-aggrecan (1:2000, Invitrogen, CA, USA), anti-phosphorylated P-38 (1:1000, Cell Signaling Technology, MA, USA), P-38 (1:1000, Cell Signaling Technology, MA, USA), anti-phosphorylated Erk1/2 (1:1000, Cell Signaling Technology, MA, USA) and Erk1/2 (1:1000, Cell Signaling Technology, MA, USA). PVDF membranes were washed with TBST buffer 3 times each time for 5 min. Then, all the samples were incubated with the secondary antibody for 1 h. Finally, the protein bands on PVDF membranes were measured by ECL (Enhanced Chemiluminescence Detection System).

To determine the expression of proteins related to the chondrogenesis pathway, we performed immunofluorescence staining analysis. BMSCs were seeded into 24-well plates at 1 × 104 cells/well and then treated with GDF-5 for 24 h in the experimental group and normal medium in the control group. Each cell sample was washed three times with PBS and fixed in 4% formaldehyde solution for 30 min, and the cell membrane was destroyed by Triton X-100 (0.5%) and then blocked with immunoblocking solution (Beyotime, Shanghai, China) for another 30 min. Finally, after three washes with PBS, the samples were incubated with anti-phosphorylated P-38 (1:200, Cell Signaling Technology, MA, USA), P-38 (1:200, Cell Signaling Technology, MA, USA), anti-phosphorylated ERK1/2 (1:200, Cell Signaling Technology, MA, USA), and ERK1/2 (1:200, Cell Signaling Technology, MA, USA) antibodies overnight at 4 °C. Immunofluorescent secondary antibodies were added and coincubated for 1 h at room temperature. After DAPI (1:200; Beyotime, Shanghai, China) staining for 15 min, phalloidin staining (1:60; staining for F-actin was performed using Beyotime, Shanghai, China) was performed for 30 min. Finally, cell images were viewed with a fluorescence microscope (Keyence, Osaka, Japan).

- 1. Effect of hybrid scaffolds on SMSC chondrogenic differentiation

The expression levels of genes related to BMSC chondrogenic differentiation in different scaffold materials were analysed. BMSCs were inoculated on tissue culture plates (TCPs), ECM scaffolds and ECM/GDF-5 scaffolds and cultured in chondrogenic medium for 14 d. BMSCs cultured in TCPs were used as the control group. Total RNA was isolated from BMSCs using the Cytosolic Total RNA Isolation Kit (Foregene, Chengdu, China). cDNA was synthesized from total RNA using P5 × RT Master Mix (Toyobo, Osaka, Japan). 2× RealStar Green Fast Mix (Genstar, Beijing, China) was used to detect chondrogenic activity. The expression of cartilage-specific genes was examined using 2× RealStar Green Fast Mix (Genstar, Beijing, China). Gene expression was normalized by the ΔΔCt method, and relative gene expression was quantified by the gene expression of the housekeeping gene GAPDH. The target gene (Col 2A1, Col 1A1, Col X, Sox 9, ACAN)-specific primary primers are shown in Table S1. Three parallel replicates were set up for each group.

- 1. Animal models

All animal experiments in the study were approved by the Institutional Animal Care and Use Committee at the PLA General Hospital. Forty skeletally mature New Zealand White rabbits (male, 2.8-3.2 kg, 6 months old) were used as animal models and randomly allocated to 4 groups, with 10 rabbits in each group: (A) the negative control group, (B) the ECM group, (C) the ECM/GDF-5 group and (D) the sham group. After adequate routine surgical preparation and provincial anaesthesia, the patella was dislocated laterally through the medial parapatellar approach of the rabbit knee joint, exposing the trochlear part fully, and then flexing the knee joint. Full-thickness cartilage defects (3.5 mm in diameter, 1.5 mm in depth) were created in the trochlear groove of the distal femur. For the negative control group, a simple defect was made. For the experimental group, scaffolds (ECM and ECM/GDF-5) were implanted into the defect, and the patella was immediately reduced after dislocation in the sham group. In the sham group, after haemostasis, the joint capsule and skin were sutured successively, and the skin incision was disinfected with iodophor. Routine antibiotics and painkillers were administered for seven days after surgery. All rabbits were allowed to freely move after surgery. After 6 or 12 weeks, all rabbits were euthanized at each time point, and the knee joints of the rabbits were sampled. After taking the samples, we observed the repair area and took general photos of the samples. Then, the images were provided to three researchers with extensive experience in cartilage regeneration, and the specific groups were unknown and scored according to the International Society for Cartilage Repair (ICRS) macroassessment guidelines (Table S2).

- 1. Micro-computed tomography (micro-CT) analysis

After the macro evaluation, the samples were placed and scanned in the General Electric (GE) Explorer Locus SP system (GE, Boston, MA, USA). After scanning, the appropriate sagittal and coronal cross sections of cartilage regeneration were adjusted in the software, 3D reconstruction was performed on each femur sample, and a columnar region of interest (ROI) (diameter 3.5 mm, height 1 mm) was selected at the sample defect regeneration area. Then, the bone mineral density (BMD) and bone volume/tissue volume (BV/TV) of the ROI of each sample were analysed. After macroevaluation by professionals, we performed CT scans of each sample using the General Electric (GE) Explorer Track SP system (GE, Boston, MA, USA) and three-dimensional reconstruction of each femoral specimen. A columnar area of interest (ROI, 3.5 mm in diameter, 1 mm in height) was selected for each defect regeneration area. The bone mineral density (BMD) and bone volume fraction (BVF) were analysed, and subchondral bone regeneration was evaluated in each group.

- 1. Histological evaluation and semiquantitative histological scoring

After micro-CT analysis, the collected four groups of femur specimens were fixed in 4% paraformaldehyde for 5 d and decalcified with 10% EDTA for 1.5 months. During this process, the specimens were continuously pruned. After decalcification and dehydration, the specimens were embedded in paraffin, sliced at 6 μm, and stained with H&E, saffron and wolverine red according to standard procedures. The immunohistochemical staining procedure for type II collagen is as follows: briefly, after section dewaxing and washing, hydrogen peroxide was used to remove endogenous peroxidase. The samples were then infiltrated with 0.5% Triton X-100. After washing with PBS, the slices were sealed and incubated with anti-collagen primary antibody (1:200, Novus, NY, USA) at 4 °C overnight. Finally, an immunohistochemical secondary antibody was added, and a chromogenic agent was used. After dehydration, neutral resin was used to seal the sheet, which was observed and photographed under a microscope. All images were provided to 2 researchers with extensive experience in cartilage histopathology and without knowledge of the groupings scored according to the O'Driscoll scoring system evaluation guidelines (Table S3).

- 1. Biomechanical testing

At 6 and 12 weeks after surgery, the mechanical condition of the regenerated tissue was evaluated. The mechanical properties of the samples were tested with a biomechanical testing machine (Bose, 5100). Young's modulus (which reflects mechanical strength) was calculated by using the following formula: E=(FL)\/(sΔL), where F is the force applied, ΔL is the variation of deformation, and S is the area of the indenter.

- 1. Biochemical testing

At the end of the biomechanical assay, the regenerated tissues were removed for the testing of biological components, and the GAG and HYP contents were examined by the GAG content DMMB colorimetric kit and the hydroxyproline assay kit. The specific steps were carried out according to the relevant instructions of the kits.

1.13 Comparison of FDM and LDM scaffolds

1.13.1 Preparation of FDM and LDM scaffolds

Polycaprolactone (PCL) scaffolds are prepared by two printing modes: fused deposition modelling (FDM) and low-temperature deposition modelling (LDM) printing. For FDM printing, biodegradable PCL polymer (Mw= 45,000, Sigma, USA) particles were placed in a bio3D printer, and then the heated and melted PCL slurry was printed through a printing nozzle according to a preset model to obtain the FDM-PCL scaffold. For LDM printing, in brief, the PCL bioink was made by taking 400 mg of PCL particles and adding them to 6 ml of acetic acid. The mixture was placed in a magnetic stirrer heated at 65 °C and stirred for 12 hours to fully dissolve, and then the slurry was centrifuged at 2000 r/min for 5 minutes to remove bubbles. The PCL slurry was returned to room temperature and transferred to a 3D printer for low-temperature deposition for printing to obtain LDM-PCL. The detailed printing procedures are described in section 2.2.1.

LDM-ECM scaffolds were prepared by low-temperature deposition printing. The method was the same as in 2.2.1.

1.13.2 Scanning electron microscopy (SEM) of the three scaffolds

After FDM-PCL, LDM-PCL and LDM-ECM scaffolds were prepared, we used an inverted microscope to observe the general structure of the scaffolds. Subsequently, the microscopic structure of the scaffold was observed by scanning electron microscopy (SEM). The detailed procedures are described in section 2.2.2.

1.13.3 Cell viability and proliferation assay of scaffolds

To evaluate the biocompatibility of the FDM-PCL, LDM-PCL and LDM-ECM scaffolds, we performed cell live/dead staining and CCK-8 assays for verification. The specific steps are described in section 2.3.2.

1.13.4 Chondrogenic differentiation assay of scaffolds

To evaluate the effects of FDM-PCL, LDM-PCL and LDM-ECM scaffolds on the chondrogenic differentiation ability of BMSCs, 1x10^6^ BMSCs were implanted on various scaffolds and cultured in chondrogenic differentiation medium for 14 days. The expression of chondrogenic-specific genes was detected by RT‒qPCR. Detailed experimental procedures are given in section 1.4 of the Supplementary Information.

1. **Supplementary Results**
   1. General view and scanning electron microscopy (SEM) of scaffolds

As shown in Figure S4, PCL scaffolds printed by FDM had a smooth appearance. However, the surface of PCL and ECM scaffolds printed by LDM was rough, and the wire diameter was evenly distributed. Then, the three scaffolds were observed by electron microscopy. The fibre surface of the FDM-PCL scaffolds was relatively flat and smooth, and almost no micropores were formed. The LDM-printed PCL and ECM scaffolds were characterized by a hierarchical porous sponge-like structure consisting of highly interconnected macropores and micropores throughout the scaffolds. In high-magnification images, the LDM-printed fibres exhibited a honeycomb appearance. Compared with LDM-PCL scaffolds, the micropores in the fibre diameter of LDM-ECM scaffolds have a larger diameter and higher connectivity.

- 1. Cell viability and proliferation assay of scaffolds

BMSCs were seeded on three different scaffolds for 7 days, and the viability of the scaffolds was observed by live/dead staining. Confocal microscopy results showed that most of the BMSCs were living cells (green), and only a few were dead (red). Moreover, it was observed that the number of cells attached to FDM-PCL scaffolds was quite limited due to the smooth surface, while the LDM scaffold group showed stronger cell adhesion, and the LDM-ECM scaffold was obviously more beneficial to cell growth (Figure S5A).

To further assess the effect of the three scaffolds on the proliferation of BMSCs, cell proliferation was assessed by CCK-8 assay (Figure S5B) after 1, 4, and 7 days of scaffold culture. The results showed that the OD values in all three stent groups increased with time, with the highest OD values in the LDM-ECM group at days 4 and 7, which was consistent with the results of live and dead staining. In conclusion, LDM-ECM scaffolds may be more beneficial to the proliferation of bone marrow mesenchymal stem cells.

- 1. Chondrogenic differentiation assay of scaffolds

The expression of chondrogenic genes in BMSCs on FDM-PCL, LDM-PCL and LDM-ECM scaffolds was detected by RT‒qPCR. As shown in Figure S5C, compared with those in the FDM-PCL group, chondrogenic genes (COL 2, SOX 9 and ACAN) of BMSCs in the LDM-PCL group were significantly upregulated during culture, indicating that the surface micropore structure, roughness and topology of LDM-PCL scaffolds could facilitate the chondrogenic differentiation of BMSCs in the same material scaffold culture environment. The expression levels of related genes in the LDM-ECM scaffold group were also significantly increased compared with those in the LDM-PCL group, which may be because ECM, a pure natural biomaterial, provides a more suitable microenvironment for the survival and differentiation of BMSCs and promotes the differentiation of BMSCs. In conclusion, the natural porous topological microenvironment provided by LDM-ECM scaffolds is more conducive to the chondrogenic differentiation of BMSCs.

**Supplementary Figures and Tables**

A


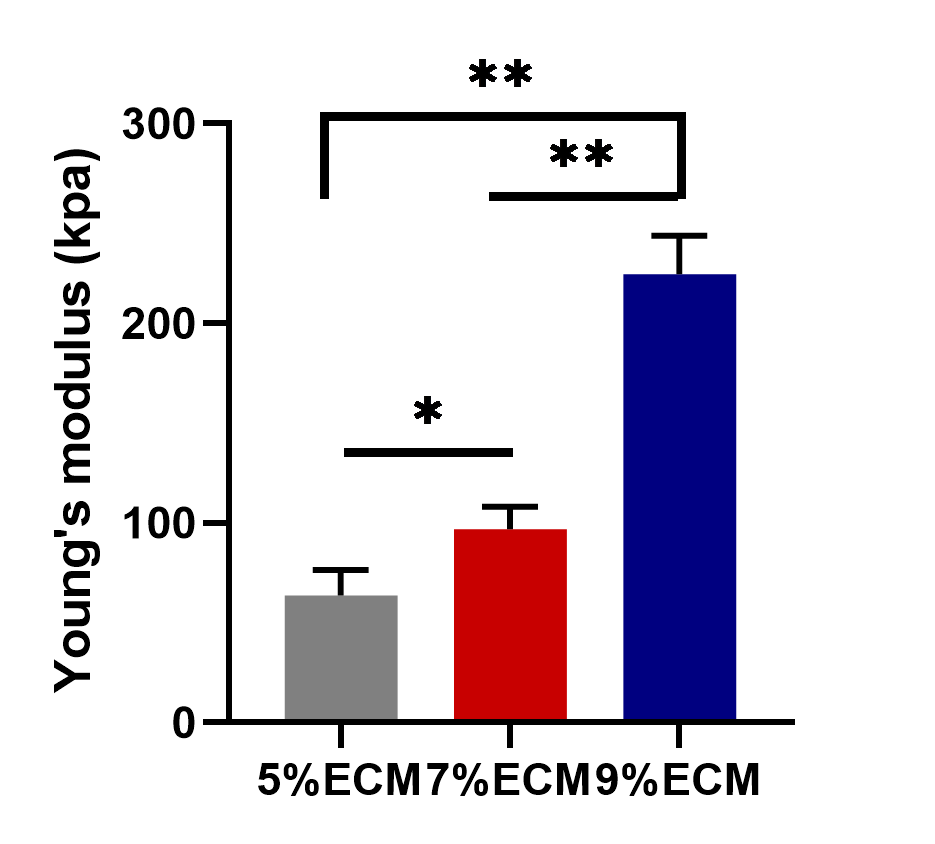

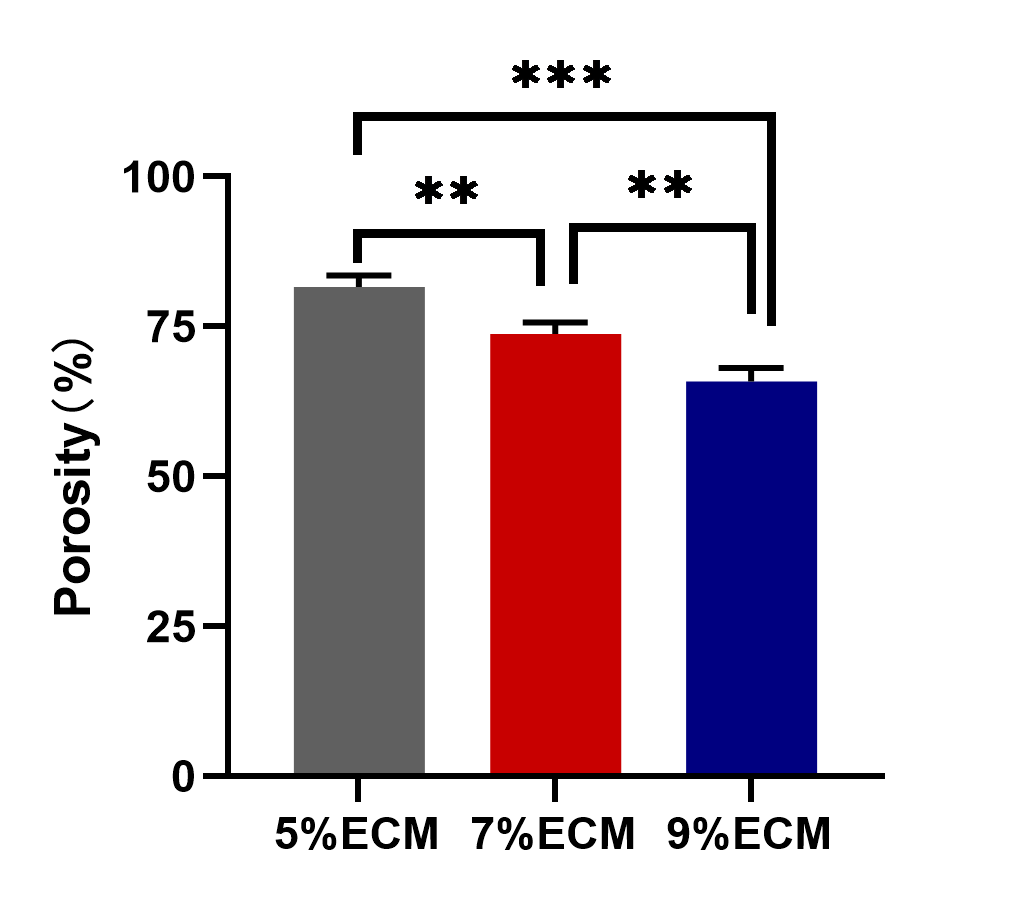


B

**Figure S1.** (A) Mechanical properties of 5%, 7% and 9% ECM scaffolds. (B) Porosity of 5%, 7% and 9% ECM scaffolds.


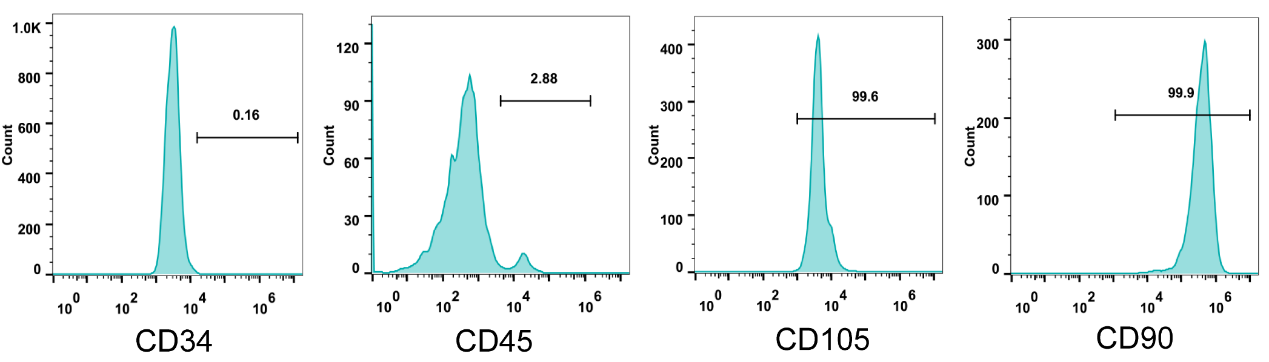


**Figure S2.** Flow cytometric analysis of MSC-specific surface markers for CD 34, CD 45, CD 90 and CD 105.


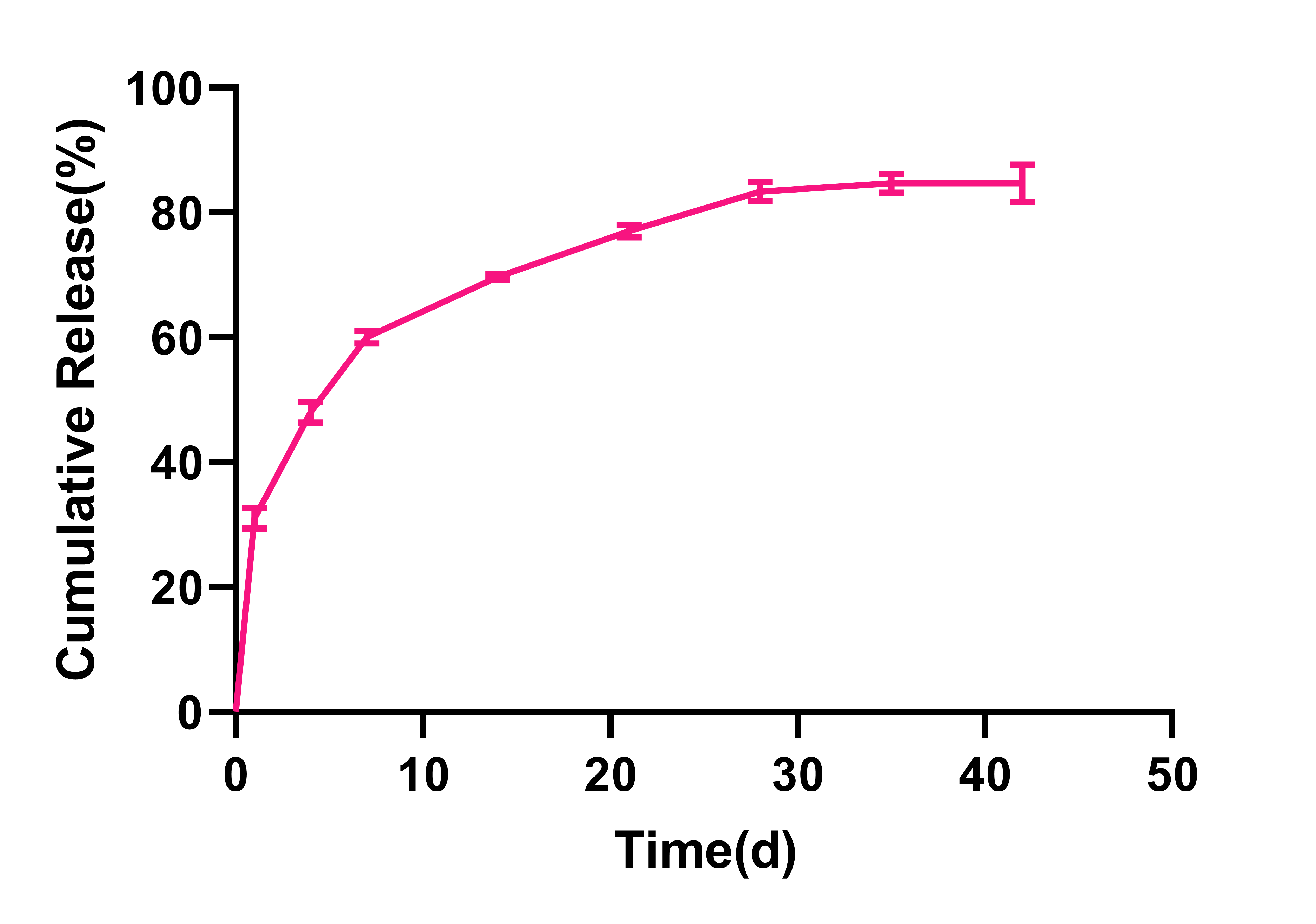


**Figure S3.** Release behaviour of ECM/GDF-5 scaffold.


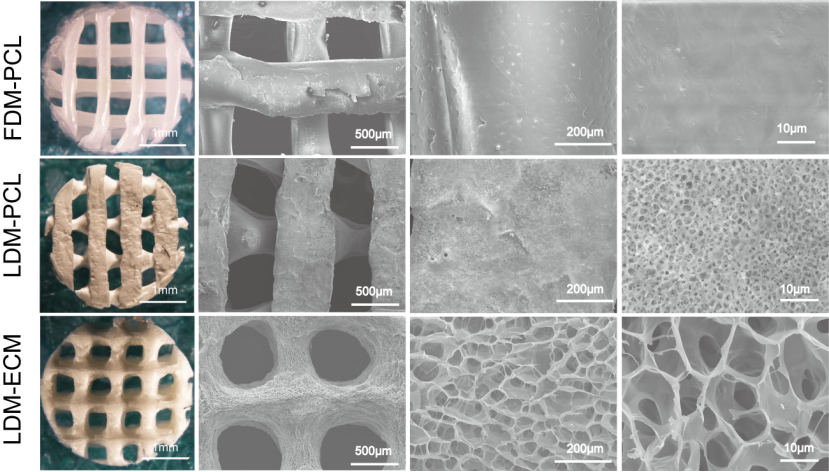


**Figure S4.** Macroscopic and SEM images of FDM-PCL, LDM-PCL and LDM-ECM scaffolds.


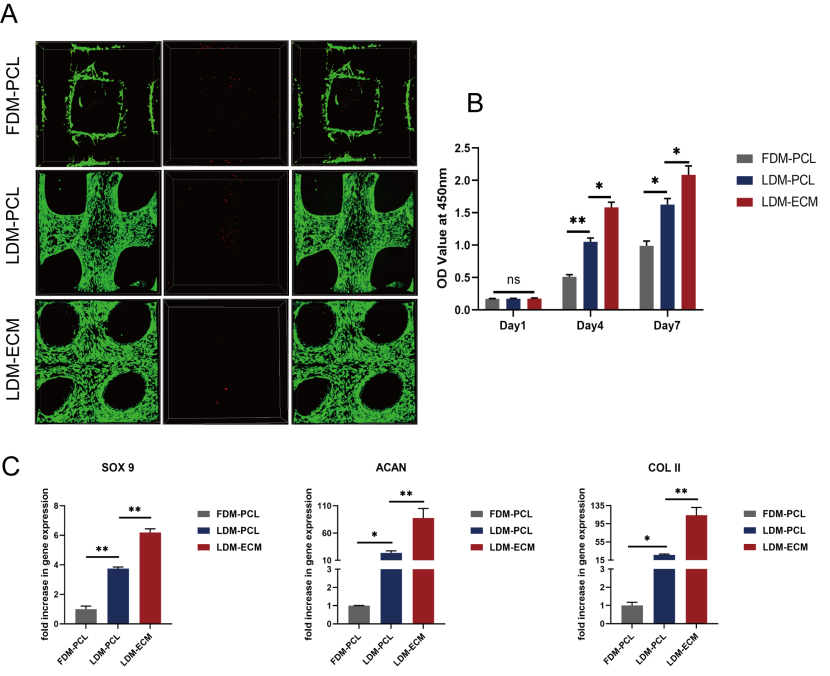


**Figure S5. Biocompatibility and chondrogenic differentiation analysis of the three scaffolds.** (A) Live/dead staining (green: live cells, red: dead cells) of BMSCs on the scaffolds. (B) CCK-8 assay results of BMSCs cultured on the scaffolds for 1 day, 4 days, and 7 days (n=4). (C) Expression of SOX 9, ACAN and Col 2A1 in the SMSCs on three scaffolds (n = 3). Statistical analysis: *p < 0.05, **p < 0.01, ***p < 0.001.

**Table S1.** Primer sequences for quantitative RT‒PCR.

| **Gene** | **Primer** | **Primer Sequence (5' to 3')** |
| --- | --- | --- |
| *SOX9* | Forward | CCAGCAAGAACAAGCCACAC |
| *SOX9* | Reverse | CTTGCCCAGAGTCTTGCTGA |
| *COL 2A1* | Forward | GAGTGGAAGAGCGGAGACTACTG |
| *COL 2A1* | Reverse | GTCTCCATGTTGCAGAAGACTTTCA |
| *Aggrecan* | Forward | CTAGCTGCTTAGCAGGGATAACG |
| *Aggrecan* | Reverse | TCATGTAGACCATGTAGTTGAGGTCA |
| *COL 1* | Forward | GTGCTAAAGGTGCCAATGGT |
| *COL 1* | Reverse | ACCAGGTTCACCGCTGTTAC |
| *COL X* | Forward | ATGGTGAGGCAGGTCCAAGAGG |
| *COL X* | Reverse | GGTTAGCACTGACAAGAGGCATCC |
| *GAPDH* | Forward | CAAGAAGGTGGTGAAGCAGG |
| *GAPDH* | Reverse | CACTGTTGAAGTCGCAG |

**Table S2.** International Cartilage Repair Society (ICRS) macroscopic evaluation guidelines.

| ICRS Cartilage Repair Assessment | | Points |
| --- | --- | --- |
| Degree of defect repair | Level with surrounding cartilage | 4 |
|  | 75% repair of defect depth | 3 |
|  | 50% repair of defect depth | 2 |
|  | 25% repair of defect depth | 1 |
|  | 0% repair of defect depth | 0 |
| Integration to border zone | Complete integration with surrounding cartilage | 4 |
|  | Demarcation border <1 mm | 3 |
|  | 3/4 of graft integrated, 1/4 with a notable border >1 mm wide | 2 |
|  | 1/2 of graft integrated with surrounding cartilage, 1/2 with a notable border >1 mm | 1 |
|  | From no contact to 1/4 of graft integrated with surrounding cartilage | 0 |
| Macroscopic appearance | Intact smooth surface | 4 |
|  | Fibrillated surface | 3 |
|  | Small, scattered fissures or cracks | 2 |
|  | Several small or few large fissures | 1 |
|  | Total degeneration of grafted area | 0 |
| Overall repair assessment | Grade I: normal | 12 |
|  | Grade II: nearly normal | 11–8 |
|  | Grade III: abnormal | 7–4 |
|  | Grade IV: severely abnormal | 3–1 |

**Table S3.** Modified O’Driscoll score system.

| Characteristic | Grading | Score |
| --- | --- | --- |
| I. Hyaline cartilage | 80%-100% | 8 |
|  | 60%-80% | 6 |
|  | 40%-60% | 4 |
|  | 20%-40% | 2 |
|  | 0%-20% | 0 |
| II. Structural characteristics |  | |
| A. Surface irregularity | Smooth and intact | 2 |
|  | Fissures | 1 |
|  | Severe disruption, fibrillation | 0 |
| B. Structural integrity | Normal | 2 |
|  | Slight disruption, including cysts | 1 |
|  | Severe lack of integration | 0 |
| C. Thickness | 100% of normal adjacent cartilage | 2 |
|  | 50%-100% or thicker than normal | 1 |
|  | 0%-50% | 0 |
| D. Bonding to adjacent cartilage | Bonded at both ends of graft | 2 |
|  | Bonded at one end/partially both ends | 1 |
|  | Not bonded | 0 |
| III. Freedom from cellular changes of degeneration | Normal cellularity, no cluster | 2 |
|  | Slight hypocellularity, <25% chondrocyte clusters | 1 |
|  | Moderate hypocellularity, >25% clusters | 0 |
| IV. Freedom from degenerate changes in adjacent cartilage | Normal cellularity, no clusters, normal staining | 3 |
|  | Normal cellularity, mild clusters, moderate staining | 2 |
|  | Mild or moderate hypocellularity, slight staining | 1 |
|  | Severe hypocellularity, slight staining | 0 |
| V. Reconstitution of subchondral bone | Complete reconstitution | 2 |
|  | >50% reconstitution | 1 |
|  | ≤50% reconstitution | 0 |
| VI. Bonding of repair cartilage to de novo subchondral bone | Complete and uninterrupted | 2 |
|  | <100% but >50% reconstitution | 1 |
|  | <50% complete | 0 |
| VII. Safranin O staining | >80% homogenous positive stain | 2 |
|  | 40%-80% homogenous positive stain | 1 |
|  | <40% homogenous positive stain | 0 |
| Total score | | Max. 27 |
